# Parsing Hippocampal Theta Oscillations by Nested Spectral Components during Spatial Exploration and Memory-Guided Behavior

**DOI:** 10.1016/j.neuron.2018.09.031

**Published:** 2018-11-21

**Authors:** Vítor Lopes-dos-Santos, Gido M. van de Ven, Alexander Morley, Stéphanie Trouche, Natalia Campo-Urriza, David Dupret

**Affiliations:** 1 Medical Research Council Brain Network Dynamics Unit, Department of Pharmacology, University of Oxford, Oxford OX1 3TH, UK

**Keywords:** hippocampus, oscillations, theta, gamma, sharp-wave/ripples, memory

## Abstract

Theta oscillations reflect rhythmic inputs that continuously converge to the hippocampus during exploratory and memory-guided behavior. The theta-nested operations that organize hippocampal spiking could either occur regularly from one cycle to the next or be tuned on a cycle-by-cycle basis. To resolve this, we identified spectral components nested in individual theta cycles recorded from the mouse CA1 hippocampus. Our single-cycle profiling revealed theta spectral components associated with different firing modulations and distinguishable ensembles of principal cells. Moreover, novel co-firing patterns of principal cells in theta cycles nesting mid-gamma oscillations were the most strongly reactivated in subsequent offline sharp-wave/ripple events. Finally, theta-nested spectral components were differentially altered by behavioral stages of a memory task; the 80-Hz mid-gamma component was strengthened during learning, whereas the 22-Hz beta, 35-Hz slow gamma, and 54-Hz mid-gamma components increased during retrieval. We conclude that cycle-to-cycle variability of theta-nested spectral components allows parsing of theta oscillations into transient operating modes with complementary mnemonic roles.

## Highlights

• Spectral profiling of single theta waves enables studying inter-cycle variability
• Theta spectral components feature different spiking patterns and ensembles
• Co-firing in theta cycles nesting mid-gamma undergo enhanced offline reactivation
• Theta components relate differently to learning and memory retrieval demands

## Teaser

Lopes-dos-Santos et al. reveal that the cycle-to-cycle variability of CA1 theta oscillations, often averaged out as biological noise, reflects dynamic tuning of hippocampal operations related to different stages of memory processing.

## Introduction

Neuronal activity in the hippocampal circuit is organized on multiple timescales by a collection of network oscillators (Buzsáki, 2010). These oscillations are typically described as rhythmic fluctuations in the local field potentials (LFPs) and correlate with behavior (Buzsáki, 2002, O’Keefe and Nadel, 1978). During active exploration, theta (5–12 Hz) oscillations dominate the hippocampal CA1 area of the rodent brain (Vanderwolf, 1969) and orchestrate neuronal firing (Csicsvari et al., 1999, Klausberger et al., 2003, O’Keefe and Recce, 1993). Theta cycles have been suggested to support the packaging of principal cell spiking into functional ensembles via the provision of discrete windows in which incoming streams of information are processed (Gupta et al., 2012, Hasselmo et al., 2002, Luczak et al., 2015, Buzsáki and Moser, 2013, Mizuseki et al., 2009, Rennó-Costa and Tort, 2017). However, it remains unclear whether these computations are consistent across theta cycles or whether they are dynamically tuned on a cycle-by-cycle basis.

A prominent feature of the hippocampal theta rhythm is its co-occurrence with bouts of faster oscillations that span the gamma (30–140 Hz) frequency band (Bragin et al., 1995, Colgin, 2015a, Csicsvari et al., 2003, Lasztóczi and Klausberger, 2014, Schomburg et al., 2014). In the hippocampal CA1 area, this broad frequency range has been subdivided into slow (∼30–50 Hz) and mid (∼50–100 Hz) gamma oscillations, which present maximum amplitude at distinct theta phases (Belluscio et al., 2012, Colgin et al., 2009, Middleton and McHugh, 2016, Scheffer-Teixeira et al., 2012, Yamamoto et al., 2014) and emerge from separate locations along the somato-dendritic axis of CA1 principal cells (Lasztóczi and Klausberger, 2016, Schomburg et al., 2014), whereas fast (∼100–140 Hz) gamma components originate from the pyramidal layer (Lasztóczi and Klausberger, 2016, Schomburg et al., 2014). These gamma band oscillations reflect the synchronous activity of distinct neuronal circuits (Bragin et al., 1995, Colgin et al., 2009, Csicsvari et al., 2003, Fernández-Ruiz et al., 2017, Lasztóczi and Klausberger, 2014) and could correspond to different network states (Carr and Frank, 2012, Colgin, 2015b). Thus, we hypothesized that the cycle-by-cycle variability of theta-nested oscillations reports flexible switching of the hippocampal network between different operating modes, such as memory encoding and retrieval.

To investigate the variability of CA1 theta oscillations on a cycle-by-cycle basis, we designed an unsupervised framework to extract the spectral content of individual theta cycles. Our analysis retrieved two spectral components consistent with slow and fast gamma oscillations, two components with main frequencies within the mid-gamma range, and one within the beta (21–23 Hz peak frequency) range. These theta-nested spectral components (tSCs) differed in their theta phase amplitude modulation and correlated with distinguishable principal cell ensembles at the single-cycle level. We consistently observed these tSCs across mice and recording paradigms and also in rat CA1 pyramidal cell layer LFPs. Furthermore, principal cell co-firing patterns within theta cycles dominated by mid-gamma oscillations underwent enhanced reactivation during subsequent sleep/rest sharp-wave/ripple (SWR; 135–250 Hz) events. Finally, we found that theta-nested 80-Hz mid-gamma oscillations were selectively strengthened during the learning stage of a goal-directed spatial task, whereas the slower (22 Hz, 35 Hz, and 54 Hz) frequency theta-nested components were stronger during the memory retrieval stage. Altogether, these findings characterize hippocampal CA1 as a versatile circuit that engages in different operating modes reflected in theta-nested spectral components during exploratory and memory-guided behavior.

## Results

We first used multichannel extracellular tetrode recordings to monitor both principal cell spiking and LFPs from the CA1 pyramidal layer of the dorsal hippocampus in mice exploring open fields.

### Profiling Individual Theta Cycles by Their Spectral Content

**Figure 1:**
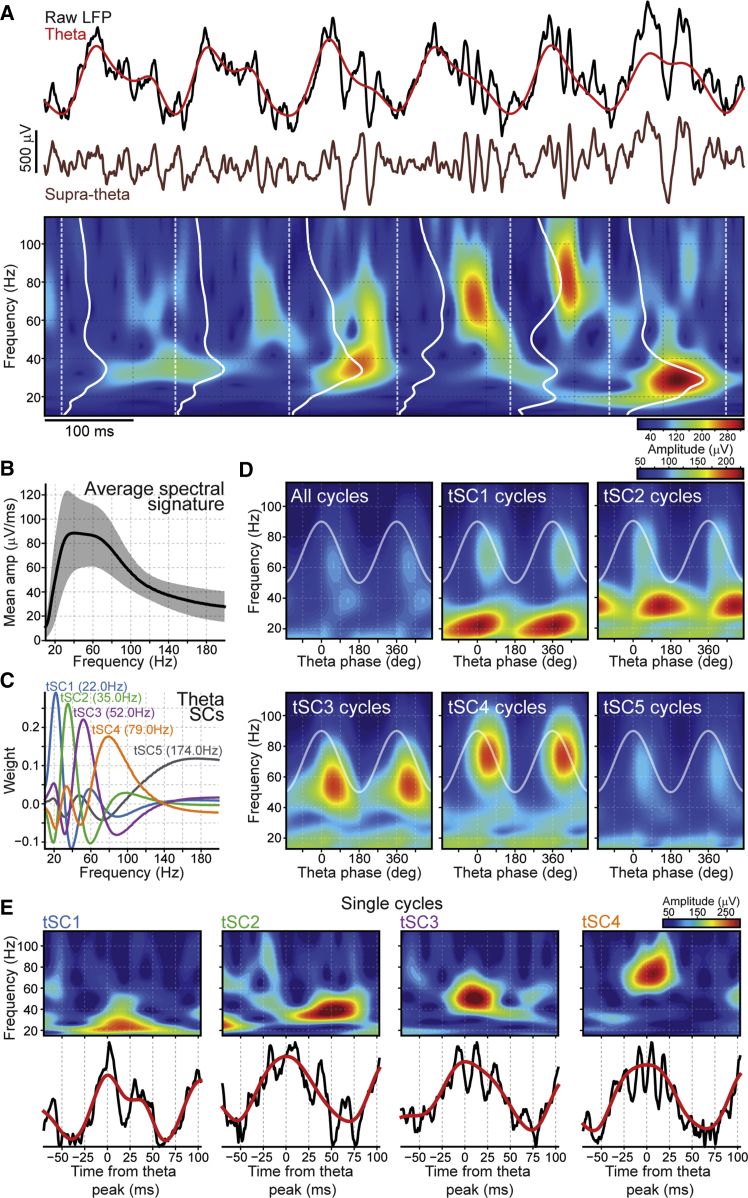
Single-Cycle Profiling of CA1 Theta Oscillations (A) Top: a raw LFP example trace (black) recorded from the CA1 pyramidal layer along with its theta (red) and supra-theta (brown) signals extracted by EEMD (see also Figure S1). Bottom: wavelet spectrogram of the supra-theta signal shown above. White dashed lines mark theta troughs. White solid traces represent single-cycle spectral signatures. (B) Average spectral signature computed using all theta cycles pooled together. The shaded area represents SD. (C) Example of theta-nested spectral components (tSCs) extracted from the spectral signatures of individual theta cycles from a recording day. Peak frequencies of tSCs for that recording day are shown in brackets. Peak frequencies across all mouse recording days (median and interquartile range) are as follows: tSC1, 22 Hz, 21–23 Hz; tSC2, 35 Hz, 34–36 Hz; tSC3, 54 Hz, 52–55 Hz; tSC4, 80 Hz, 77–82 Hz; tSC5, 169 Hz, 153–174 Hz. See Figures S2A, S2B, S2I, S2J, and S8B for the other mouse recording days and Figure S2D for the rat dataset. Note that, to subsequently quantify the strength of a given tSC nested in an individual theta cycle, we projected the spectral signature of that cycle onto the axis defined by that tSC’s weight vector (see STAR Methods for details). This projection represents a measure of similarity between a single-cycle spectral content and a given tSC. (D) Mean amplitude of supra-theta frequencies computed from the raw LFPs as a function of theta phase for the example recording day shown in (C). Each panel displays this analysis computed either for all theta cycles together (see also Figure 2E) or selectively for theta cycles strongly expressing a particular tSC (i.e., above the tSC threshold; see also Figure S4). Cosine indicates theta phase reference with two cycles for clarity. See also Figure S3B for frequencies above 110 Hz and Figure S5A for the amplitude of supra-theta frequencies as a function of theta phase in cycles with weaker tSC strength. (E) Raw LFP spectrogram (top) along with the raw LFP and theta signal of single-cycle examples drawn from the tSC cycles displayed in (D). See also Figure S4A.

We aimed to characterize the spectral signature of individual theta cycles observed in LFPs of the CA1 pyramidal cell layer. To do so, we developed an unsupervised framework that identifies transient oscillations nested within theta cycles (Figure 1). First, we applied ensemble empirical mode decomposition (EEMD) to break down raw LFPs into their theta and supra-theta signals (Figures 1A and S1). Unlike linear filters, the EEMD is suited to non-stationary signals and allows instantaneous frequencies to follow asymmetrical waveforms (Wu and Huang, 2009), as seen in theta oscillations (Figure 1A). Next, we computed the spectrogram (from 10 to 200 Hz) for the supra-theta signal and calculated its local mean within the boundaries of each theta cycle. As a result, each theta cycle was associated with a curve carrying the amplitude of different frequencies within that cycle (Figure 1A). We refer to these power spectrum-like curves as spectral signatures.

We observed a large cycle-by-cycle variation in these spectral signatures (Figure 1A), which was hidden when only considering the averaged spectral signature from all theta cycles pooled together (Figure 1B). We then applied independent component analysis to these spectral signatures to extract, in an unsupervised manner, frequency components representing distinct signals that consistently occur across theta cycles. We found that each resulting tSC attributed large weights to different frequency bands (Figure 1C), some of which were consistent with CA1 oscillations described previously (Belluscio et al., 2012, Colgin et al., 2009, Lasztóczi and Klausberger, 2016, Middleton and McHugh, 2016, Scheffer-Teixeira et al., 2012, Schomburg et al., 2014, Sirota et al., 2008). Notably, tSC2 presented larger weights around 35 Hz, matching the central frequency of slow gamma oscillations. The next two components, tSC3 and tSC4, exhibited peaks within the mid-gamma band, around 54 Hz and 80 Hz, respectively. tSC5 was dominated by frequencies above 100 Hz, consistent with fast gamma activity. Finally, tSC1 attributed larger weights to frequencies around 22 Hz. All of these tSCs were robustly detected across recording experiments and animals (n = 20 recording days from 10 mice; Figures S2A, S2B, and S3A). Interestingly, the strength of tSCs presented a sharply decaying autocorrelation from one theta cycle to the next (Figure S2C). Moreover, we retrieved similar tSCs by performing the same analysis on LFPs recorded from the rat CA1 pyramidal layer (Figure S2D).

We next assessed whether the amplitude of the oscillations extracted by tSCs was modulated by the ongoing theta phase. For each theta phase, we computed the mean amplitude of a wide range (10–200 Hz) of frequencies from the raw LFP by either using all cycles or only those strongly expressing a given tSC (Figures 1D, S2E, and S3B). Cycles with strong tSC2 exhibited prominent slow gamma oscillations with increased amplitude along the descending phase of theta, close to its trough; whereas the tSC3 and tSC4 cycles showed increases in mid-gamma amplitudes just after the theta peak. tSC5 cycles did not show prominent components in frequencies below 100 Hz but in the fast gamma range, with increased amplitude at the theta trough (Figure S3B). Finally, tSC1 cycles showed a strong, ∼22-Hz component with maximum amplitude at theta peaks. Although no such hippocampal beta band component was previously reported to be coupled to theta oscillations, visual inspection of the raw signal confirmed that tSC1 cycles were marked by a substantial deflection around the theta peak, indicating that this signal was not a theta harmonic artifact (Figures 1E and S4A). On average, 36.3% of theta cycles contained at least one strong tSC.

The magnitude of hippocampal gamma oscillations correlates with speed in rodents. Accordingly, we found that the strength of every tSC was positively correlated with mouse speed (Figure S2F), consistent with previous work on CA1 gamma oscillations in mice (Chen et al., 2011), with tSC3 being the least speed-modulated and tSC4 the most (all p < 0.0002, bootstrap test). Interestingly, the strength of both rat tSC1 and tSC2 was negatively correlated with speed (Figure S2G), in line with previous work on rat slow gamma oscillations (Ahmed and Mehta, 2012, Kemere et al., 2013).

Altogether, these results reveal that theta cycles can be profiled by their transient spectral content and that single-cycle spectral signatures greatly differ from the grand average. Our spectral decomposition also showed that tSC strengths of individual theta cycles lie on a multidimensional continuum rather than clustering into non-overlapping subsets (Figure S4B). This suggests that theta-nested oscillations are weighted in each cycle rather than being expressed in a binary fashion.

### Theta-Nested Spectral Components Are Associated with Different Firing Modulation of Principal Cells

**Figure 2:**
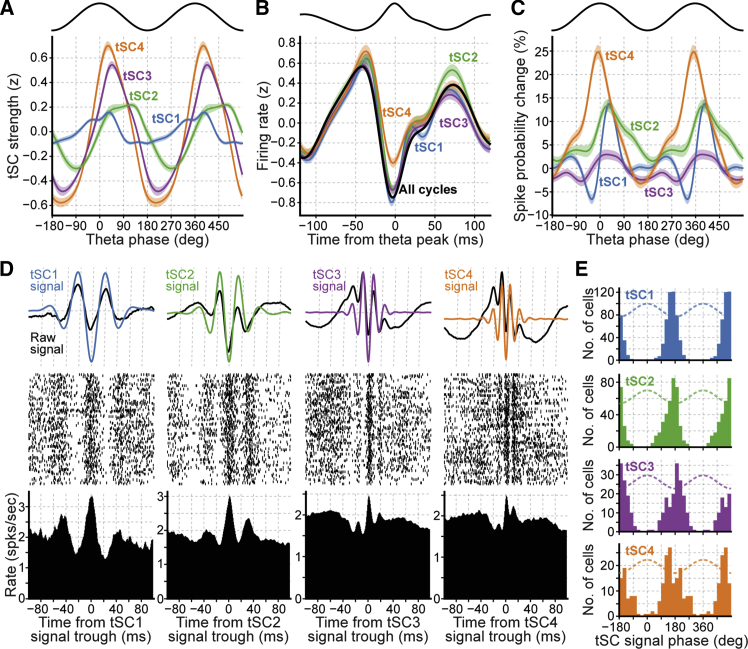
Single-Cycle Principal Cell Firing Differs According to Expression of Theta-Nested Spectral Components (A) Strength of tSCs as a function of theta phase. Each color-coded curve represents the strength computed at each time point as the inner product between the corresponding tSC and the supra-theta signal spectrogram. Theta-nested SC1 strength peak theta phase: median 35°; interquartile range, 30°–41°; tSC2, 121°, 46°–131°; tSC3, 42°, 35°–50°; tSC4, 34°, 27°–41°; n = 20 recording days from 10 mice. Shaded areas indicate SEM. Cosine indicates theta phase reference with two cycles for clarity. (B) Average instantaneous firing rate (*Z*-scored) of principal cells in theta cycles. Results were computed either using all theta cycles or only those strongly expressing a given tSC. Results shown as means over all principal cells, with shaded areas indicating 95% confidence interval. The averaged raw LFP waveform is displayed on top as a reference. (C) Change in spike probability of principal cells as a function of ongoing theta phase. Changes were computed for cycles strongly expressing a given tSC and relative to the grand average (mean ± SEM). Relative spike probabilities were calculated individually for each neuron before averaging. Cosine indicates theta phase reference with two cycles for clarity. See Figures S5B and S5C for spike probability in theta cycles with weaker tSC strength. See Figure S5F for comparison with results obtained with the application of linear filters using frequency bands matching tSC main frequencies. (D) Example population principal cell firing triggered by the troughs of tSC signals. Top: average raw LFPs and tSC signals triggered by the troughs of the latter. Troughs of each tSC signal were detected within its corresponding theta cycles. A single tSC signal trough (with the most negative value) per theta cycle was used to avoid auto-correlation distortions. Center: raster plots showing the spike trains of all principal cells recorded that day. Note, in each row, the spike times (ticks) around the trough of the tSC signal detected within a theta cycle. Bottom: mean instantaneous firing rate around the troughs of the tSC signal detected in the theta cycles strongly expressing that tSC. (E) Distribution of the preferred firing phase of principal cell spikes to tSC signals. Cosines (dashed) indicates tSC signal phase reference using two cycles for clarity. Only cells with significant coupling are included. Note that the spike phase coherence to the tSC signal was greatly diminished in theta cycles nesting weaker tSC (i.e., subthreshold strength; see Figures S4B, S5D, and S5E).

In line with the previous analysis (Figure 1), we found that the relationship between the strength of each tSC and theta phase was robust across the whole mouse dataset (Figures 2A and S3C). We next investigated whether tSCs were associated with different firing modulation of principal cells (n = 1,003) during the course of theta cycles.

We first evaluated whether neuronal firing was altered within theta cycles strongly expressing a given tSC (Figures S4B, S4C, and S5A). As a general rule, the principal cell firing rate was minimal at theta peaks and higher around theta troughs (Figures 2B and S3D). However, we found that principal cell firing around theta troughs was significantly higher in tSC2 cycles compared with any other tSC (all pairwise comparisons p < 0.0072, Wilcoxon signed-rank tests). In contrast, principal cell firing around theta peaks was the highest in tSC4 cycles (all pairwise comparisons p < 0.0004, Wilcoxon signed-rank tests). These differences in instantaneous firing rate were confirmed by evaluating, for each theta phase, the spike probability observed in cycles strongly expressing each tSC relative to what was observed for all cycles (Figure 2C). Indeed, principal cell spike probability exhibited a sharp increase at the peak of tSC4 cycles compared with the grand average cycle (from −49° to 49° theta phase; multiple regression ANOVA model, controlling for animal identity) but presented a sustained increase from the descending phase to the trough for tSC2 cycles (from 0° to 103° theta phase). Principal cell spike probability fluctuated far less in tSC3 cycles. The change in spike probability was biphasic around the peak of tSC1 cycles (significantly decreased from −48° to −6° and increased from 18° to 55°). Importantly, the magnitude of such spiking modulation (Figure 2C) drastically diminished in theta cycles nesting weaker tSCs (Figures S5B and S5C).

We then evaluated whether principal cell firing in each theta cycle was further modulated at a finer temporal scale matching the corresponding tSC main frequencies. We indeed observed that principal cell spikes were also phase-coupled to tSC signals (Figures 2D and S3E–S3I). As a general rule, spike discharge was organized around the troughs of each tSC signal (Figures 2E and S3E). This spike-phase coherence to tSC signals substantially decreased in theta cycles nesting weak tSCs (Figures S5D and S5E).

Overall, these results demonstrate that theta cycle-by-cycle spectral variability has clear population-level spiking correlates.

### Theta Spectral Components Correlate with Distinguishable Neuronal Ensembles

**Figure 3:**
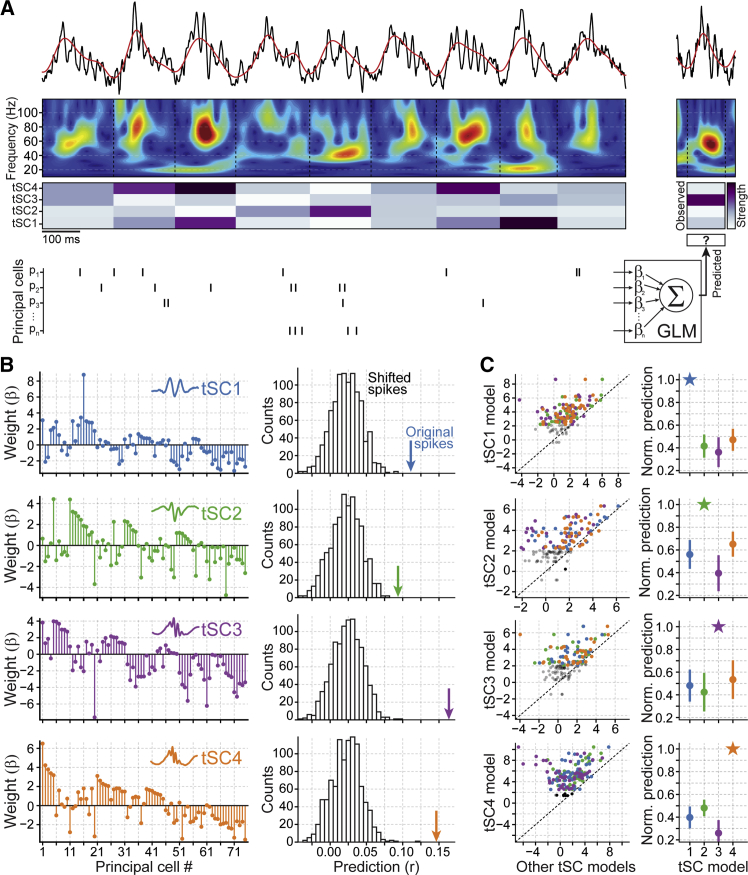
Theta-Nested Spectral Components Are Associated with Different Principal Cell Ensembles (A) The relationship between principal cell ensembles and the strength of tSC signals was assessed using generalized linear models. Each GLM was fitted to predict the strength of a given tSC in individual theta cycles from principal cell spike counts. Note that, in these analyses, GLMs are non-categorical, and the strength of each tSC signal was directly assessed on a cycle-by-cycle basis using all theta cycles. The 10-fold cross-validation procedure consisted of splitting the whole set of detected theta cycles as training (90%) and testing cycles (10%), standardizing (*Z* score) tSC strengths from all cycles by their mean and SD in the training set, fitting the GLM model to the training set, and evaluating the model on the test data. These steps were repeated iteratively until all cycles were used in the training and testing sets (see STAR Methods). (B) Left: example weight vectors containing the contribution of each principal cell (mean β coefficients across 10 cross-validated GLMs) fitted to predict the strength of a given tSC. Right: the prediction achieved by the original model is contrasted with the distribution of shift control predictions. Prediction was quantified as the Pearson correlation between observed and predicted tSC strengths. (C) Left: prediction of the strength of a given tSC by the original model (vertical axis) compared with that by the other tSC models (horizontal axis); predictions are expressed as the number of SDs away from the mean of their corresponding control distributions to compare tSC models across recording days with different numbers of cycles and neurons. Each original tSC model is represented by three dots (color-coded according to the other tSC models; dots of non-significant original models are displayed in gray). Right: predictions (mean ± 95% confidence interval) obtained from the GLMs fitted for the different tSCs. Note that GLM predictions were normalized by their original value (i.e., GLMs predicting same tSC for which they were fitted) for visualization purposes only, to make performance loss across tSC models explicit. See also Figure S6C for speed control.

Next we investigated whether each tSC was associated with particular ensembles of principal cells. For each exploration session, we trained generalized linear models (GLMs) to predict the strength of a given tSC on a cycle-by-cycle basis from the spike counts of principal cells (Figure 3A; mean principal cells per GLM, 39.25; interquartile range, 25–55). To avoid overfitting, GLMs were trained in 90% of all recorded theta cycles and tested in the remaining 10%. This was performed iteratively so that all theta cycles were tested by the end of the procedure (10-fold cross-validation). Each GLM consisted of a set of regression weights measuring the contribution of each principal cell when predicting the strength of a given tSC (Figure 3B, left). To assess the statistical significance of each tSC GLM, we repeated this procedure after shifting the original spikes across theta cycles. This control preserved the distributions of spike counts per cycle as well as the auto-correlation of each cell and the cross-correlations between them but destroyed the original relations between ensemble activity and tSC strength. We observed that the tSC strength predictions obtained by each original GLM were significantly better than those obtained from the GLM shift controls (Figure 3B, right), indicating that each tSC was associated with the activity of a particular principal cell ensemble. We also noted that the weights attributed to individual neurons by tSC GLMs were positively correlated to their speed modulation; that is, principal cells with an increased firing rate at higher speeds were, in general, more positively correlated with tSC strength (Figure S6A). Of note, the relationship between speed modulation of individual neurons and their GLM weights was the highest in tSC4 models and the weakest in tSC3 models (Figure S6A). We did not find any significant differences across tSCs between single-neuron GLM weights and other firing properties, such as spatial information (Figure S6B).

Previous studies (Ahmed and Mehta, 2012, Chen et al., 2011, Kemere et al., 2013, Zheng et al., 2015) and our own data (Figure S2F) showed that speed is an important covariate of hippocampal gamma oscillations. Because the CA1 principal cell firing rate is also correlated with speed (McNaughton et al., 1983), we tested whether the information conveyed by principal cells and extracted by the GLMs was a by-product of the co-modulation of tSC strength and spike discharge by speed. First, we found that the GLMs fitted to predict tSC strength from the original spikes and speed significantly increased the prediction over the GLMs fitted with original speed but spike content of other theta cycles (Figure S6C). Further, we found that the tSC prediction was still significant when the GLMs were re-computed using only theta cycles within a narrow range of speed values (tSC1, p < 7.6 × 10^−6^; tSC2, p < 7.1 × 10^−6^; tSC3, p < 0.0013; tSC4, p < 3.1 × 10^−6^; Wilcoxon test, comparing actual prediction values against expected from shuffled data; using cycles within 2 cm/s speed ranges: 1–3, 3–5, 5–7, 7–9, and 9–11 cm/s). These two complementary control analyses show that the information conveyed by principal cell firing about the strength of each tSC was mostly not redundant to speed information.

We further evaluated the selectivity of the relationship between the ensembles of principal cell spikes and their corresponding tSC by using the original GLMs obtained for a given tSC to predict the strength of the other tSCs. We found that the predictions for a tSC declined when using the regressions fitted with the other tSCs (Figure 3C). These results show that the spike content of individual theta cycles can predict tSC strength.

### Co-firing Patterns in Theta Cycles Nesting Mid-gamma Oscillations Undergo Enhanced Offline Reactivation

**Figure 4:**
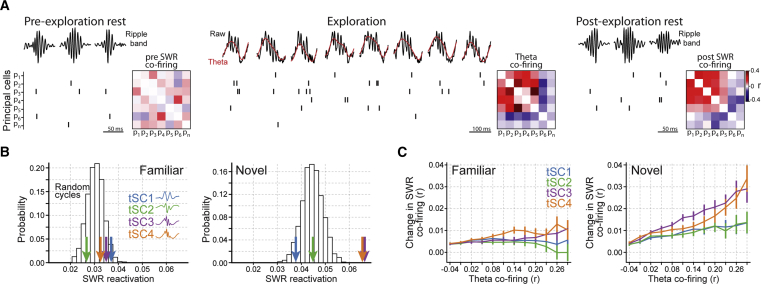
Enhanced SWR Reactivation of tSC3 and tSC4 Co-firing Patterns (A) SWR reactivation of waking patterns formed by principal cell theta co-firing. SWR reactivation was estimated by comparing the tendency of principal cell pairs to co-fire in theta cycles of a given tSC during exploration (theta co-firing) with the tendency to co-fire in SWRs during the following rest (post-SWR co-firing), controlling for their baseline co-firing in the rest before (pre-SWR co-firing). Co-firing was quantified as Pearson correlation (*r*). (B) SWR reactivation following exploration of familiar and novel environments. The SWR reactivation was measured by the coefficients of the linear regressions that predict post-SWR co-firing from theta co-firing, controlling for pre-SWR co-firing. The two histograms show the probability distributions of SWR reactivation obtained using randomly selected theta cycles from the familiar and novel environments. Color-coded arrows show the SWR reactivation obtained for theta cycles of a given tSC. Interquartile range of SWR events in post-sleep sessions: familiar interquartile range (IQR), 704–1,632; novel IQR, 701–1,758. See also Figures S7B and S7C for SWR reactivation computed from speed- and location-matched theta cycles. The dataset includes n = 18 familiar enclosure sessions and 12 novel enclosure sessions, with mice exposed both to a familiar and a novel enclosure on most of the recording days. (C) Change in SWR co-firing (from pre- to post-exploration; mean ± SEM) as a function of theta co-firing in familiar and novel environments. For each tSC, the data points correspond to cell pairs whose theta co-firing exceeded the corresponding value on the horizontal axis.

We evaluated the relation between tSCs and the CA1 neuronal dynamics of non-exploratory behavior. Previous work showed that the firing associations formed between co-active cells during exploration are later replayed during sleep/rest SWRs, presumably in support of memory consolidation (Girardeau et al., 2017, Kudrimoti et al., 1999, Peyrache et al., 2009, Rothschild et al., 2017, Wilson and McNaughton, 1994). We tested whether theta cycles were differentially associated with offline reactivation depending on their spectral signatures. To evaluate SWR reactivation, we recorded rest epochs before and after the exploration of familiar and novel environments (Figure 4A). We then compared the tendency of principal cell pairs (n = 31,722) to co-fire in cycles of a given tSC (theta co-firing) with their tendency to co-fire in SWRs of the following rest period (SWR co-firing). For all tSCs, the waking firing patterns of both familiar and novel environments were reactivated in the following rest, as shown by the significant and positive correlations between each tSC co-firing pattern and the subsequent SWR co-firing (Figure 4B; all p < 0.0001; linear regressions, controlling for pre SWR co-firing). Further, tSC co-firing patterns from novel environment exploration were more strongly reactivated than co-firing patterns from the familiar environment (Figure 4B; familiar versus novel random cycle distributions, p = 0.0046; bootstrap test). Importantly, tSC3 and tSC4 co-firing patterns of novel environments predicted SWR co-firing substantially better than co-firing patterns from randomly selected theta cycles (Figure 4B; tSC3 and tSC4 p < 0.0004; theta cycle permutation test). This was not the case for tSC1 and tSC2 cycles (both p > 0.48). The SWR reactivation strength of tSCs from familiar environments was similar to randomly selected theta cycles. Because tSCs exhibited different levels of speed modulation (Figure S2F), and because the speed modulation of gamma oscillations is stronger during novelty (Kemere et al., 2013), we tested whether the speed distribution of theta cycles could explain the observed differences in reactivation. We confirmed that the reactivation levels associated with tSC3 and tSC4 from novel environments were significantly higher compared with randomly selected cycles with the same speed and location distributions (Figure S7).

The firing associations reactivated during SWRs reflect preceding waking experience. That is, the greatest co-firing increase in SWR events occurs between cells that had fired together the most in recent theta epochs (O’Neill et al., 2008). We investigated to what extent co-activation of principal cells in theta cycles expressing particular tSCs could explain increases in SWR co-firing. More specifically, we fitted a linear regression model to predict the change in SWR co-firing (pre- to post-exploration rest) from the theta co-firing calculated from the cycles of each tSC. For the familiar environment, we found that the change in SWR co-firing only weakly related to the amount of waking co-firing in all tSCs (Figure 4C). In contrast, for the novel environment, cell pairs drastically increased their SWR co-firing as a function of both tSC3 and tSC4 co-firing (tSC3 and tSC4 β coefficients, 0.045 ± 0.007 and 0.035 ± 0.007, both p < 5.5 × 10^−8^), whereas both tSC1 and tSC2 co-firing only marginally predicted subsequent SWR firing associations (tSC1 β = 0.010 ± 0.006, p = 0.099; tSC2 β = 0.014 ± 0.006, p = 0.015).

These complementary analyses show that co-firing patterns observed in tSC3 and tSC4 cycles (i.e., the ones dominated by 54-Hz and 80-Hz mid-gamma oscillations) are the most reactivated in the subsequent sleep/rest SWRs. This enhanced reactivation was seen for the exploration of novel but not familiar environments and could not be explained by speed and location distributions.

### Current Source Density Analysis Reveals Laminar Profiles of tSC Signals

**Figure 5:**
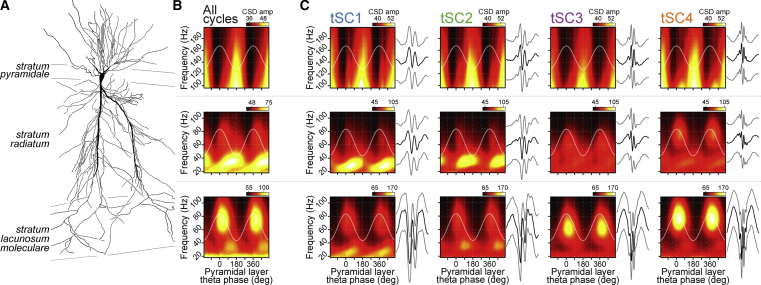
Laminar Profile of tSC-Related Currents (A) CA1 pyramidal cell reconstruction used as reference (courtesy of P. Somogyi and T. Klausberger; adapted from Lapray et al., 2012). (B) Mean amplitude of frequencies computed from the raw CSD signals as a function of theta phase using all theta cycles (theta phase defined from pyramidal layer LFP). (C) Same as in (B) but for theta cycles strongly nesting a given tSC, as detected from the pyramidal layer LFP. Raw LFPs recorded from different depths were averaged around tSC signal troughs and displayed next to a panel of the corresponding layer (black traces, with a thicker trace showing the electrode used for the amplitude-theta phase plot). See also Figures S2I and S2J for tSCs extracted from silicon probe recordings of mouse hippocampal CA1 LFPs.

To gain further insights regarding the possible contribution of each tSC to hippocampal processing of mnemonic information, we evaluated tSC laminar profiles in terms of current source densities. We extracted tSCs from LFPs recorded from the mouse hippocampal CA1 using a silicon probe spanning the somato-dendritic axis of principal cells (Figures S2H–S2J). This allowed estimating current source density (CSD) signals from different CA1 layers. We then calculated the theta phase relationship of the frequency components of these CSD signals relative to CA1 pyramidal layer theta oscillations (Figure 5) by considering either all recorded cycles (Lasztóczi and Klausberger, 2014; Figure 5B) or only those with strong tSCs (Figure 5C). We found that tSC1- and tSC2-related oscillations presented stronger *radiatum* layer currents compared with tSC3 and tSC4, which presented prominent currents in the *lacunosum moleculare* layer (Figure 5C). These observations are consistent with previous work indicating that mid-gamma CA1 oscillations are mainly generated in the *lacunosum moleculare* layer, whereas slow gamma oscillations relate more to the *radiatum* layer (Bragin et al., 1995, Lasztóczi and Klausberger, 2014, Schomburg et al., 2014).

### The Strength of tSCs Differently Relate to Stages of a Spatial Memory Task

**Figure 6:**
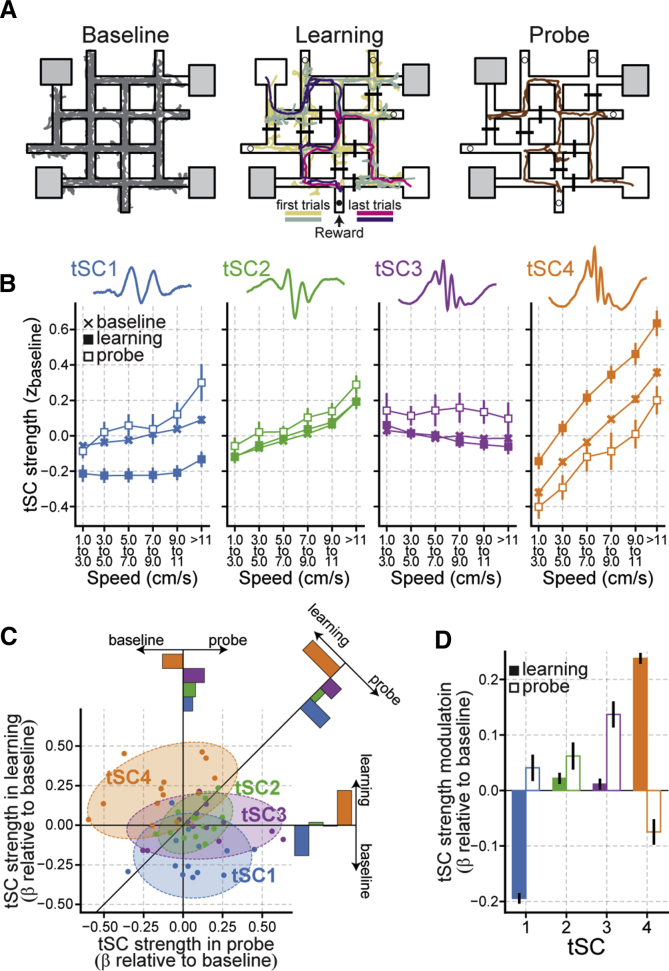
Differential Modulation of tSCs during Learning and Probe Stages of a Spatial Memory Task (A) Outline of the crossword maze task, illustrated by an example recording day. Shown are the animal’s path for each task stage, the reward location in use during learning (the black dot represents the baited plastic cap) and the four unrewarded ends of track (plain circles represent non-baited plastic caps) that day; on each task stage of that day, unused departure boxes are shaded. See also Figure S8. (B) Strength of tSCs as a function of task stage and animal speed (mean ± SEM across recording days). For each day, the strength of each tSC was averaged across all theta cycles occurring within the indicated speed range (horizontal axis). Strength values were *Z*-scored with respect to baseline (i.e., SD and mean for normalization were taken from baseline theta cycles). Data were averaged across all theta cycles within each recording day, and then grand averages were calculated from those. (C) tSC modulation during learning and probe stages. Each dot represents the tSC modulation during learning (vertical axis) and memory probe (horizontal axis) for a given recording day and relative to the baseline. The diagonal solid black trace shows the y = x line (i.e., along which the strength of a tSC in learning and probe test would be equal). Each bar plot represents the average projection of the data of each tSC on a given axis. (D) tSC modulation by task stages. tSC strength modulation was quantified relative to baseline using the coefficients from an ANOVA model controlling for speed and inter-recording day variability. Error bars represent 95% confidence interval. See also Figure S8.

We finally asked whether tSCs were differentially modulated during the behavioral stages of a memory task. We used hippocampal CA1 LFP recordings from additional mice trained to adjust, every day, their spatial knowledge of a crossword-like maze to reach a reward location (Figures 6A and S8A; n = 13 recording days from 6 mice) (McNamara et al., 2014, Tolman and Honzik, 1930). Each day started by allowing mice to explore the maze in its plain configuration (i.e., without intra-maze barriers or rewards; Figure 6A, baseline). This session was used to extract tSCs (Figure S8B) and functioned as a baseline to assess changes in tSC strength during the subsequent learning and memory retrieval stages. We next inserted a new set of intra-maze barriers and selected two departure boxes and a food reward location. Mice were then trained to learn (up to 20 trials) the most efficient path to get to the reward from the departure boxes in use that day (Figure 6A, learning; Figure S8A). Mice were further tested 1 hr after the end of learning during a memory probe test (Figure 6A, probe; Figure S8A).

We found that tSCs were differently affected across task stages. The strength of tSC4 strongly increased during learning relative to baseline (Figures 6B–6D; p < 1 × 10^−36^, ANOVA model controlling for animal speed and inter-recording day variations; Figures S8C and S8D). This tSC4 strengthening was accompanied by a strong decrease in tSC1 strength (Figures 6B–6D, p < 1 × 10^−36^). In contrast, tSC4 strength decreased during the memory probe test relative to baseline (Figures 6B–6D; p < 1.4 × 10^−12^). All remaining tSCs were strengthened during the probe test (tSC1, p = 0.0002; tSC2, p < 4.7 × 10^−8^; tSC3, p < 3 × 10^−36^), with tSC3 presenting the highest increase (Figure 6D; p < 1 × 10^−10^, all pairwise comparisons). These changes in tSC strength were consistently observed across days (Figure 6C). Because the proportion of theta cycles happening at different speeds and locations can differ across task stages (Figure S8E), we also ran a matching control to compare tSC strength across different stages, restricting the analysis to speed- and location-matched theta cycles (Figure S8F). We found that the observed task stage-related changes in tSC strength held even when controlling for speed and spatial location (Figure S8G).

When considering the learning stage, we found a strengthening of tSC4 (and tSC2) from early to late trials and the opposite trend for tSC1 (Figure S8H). Further, learning-related tSC4 enhancement was more pronounced near the goal location compared with the departure zones or near the intra-maze barriers, whereas, during the memory test, the goal and the departure zones were associated with a drastic increase in tSC1 and tSC2, respectively (Figure S8I). No such differences between the departure, the barriers, and the goal zones were seen during the baseline session, when such zones were not yet meaningful to the task (Figure S8I).

These results show that the behavioral stages of a spatial memory task affect tSCs differently. More specifically, the theta-nested 80-Hz mid-gamma component is strengthened during learning, whereas the beta, slow gamma, and 54-Hz mid-gamma components are enhanced during memory testing.

## Discussion

In this study, by introducing an unsupervised framework for single-cycle analysis of theta oscillations, we show that spectral components transiently nested in individual cycles relate to distinct spiking dynamics and distinguishable ensembles of principal cells. Further, co-firing patterns expressed in theta cycles with strong *lacunosum moleculare* layer-related oscillations are more reactivated in sleep/rest SWRs following exploration of novel environments. Finally, we found that theta-nested spectral components are differently altered by behavioral stages of a spatial memory task. Taken together, our results support the idea that theta cycle-to-cycle spectral variability reflects distinct hippocampal CA1 network operations with different contributions to memory processes.

### Single-Cycle Spectral Decomposition of Theta Oscillations Reveals Transient Tuning of CA1 Spiking

During exploratory behavior, the spiking activity of hippocampal CA1 neurons and their inputs are coupled to the phase of the ongoing theta rhythm. Medial entorhinal cortex layer III (EC3) principal cells project to the CA1 *lacunosum moleculare* layer and exhibit their highest discharge probability around the peak of the theta cycle; hippocampal CA3 pyramidal cells project to the CA1 *radiatum* layer and exhibit their highest spiking activity along the descending phase of CA1 pyramidal layer theta (Desmond et al., 1994, Ishizuka et al., 1990, Mizuseki et al., 2009, Somogyi and Klausberger, 2005, Witter et al., 2000). These inputs are integrated in the CA1 dendritic arbor and are modulated by diverse interneuron types, which also fire at particular phases of the theta cycle (Klausberger and Somogyi, 2008). Local computations then shape the expression of cell assemblies around the trough of the theta cycle (Mizuseki et al., 2009, Somogyi et al., 2013). The renewal of this set of events in each theta cycle lays the foundation of our current understanding of CA1 theta dynamics. However, it remained unclear whether theta-nested operations occur regularly from one cycle to the next or whether they are dynamically tuned on a cycle-by-cycle basis.

Here we profiled theta cycles by using their nested signals to evaluate cycle-by-cycle variations in the hippocampal circuit. We developed a single-cycle analysis framework to extract transient spectral components embedded in theta oscillations recorded from the CA1 pyramidal cell layer. We found that theta cycles holding strong tSC signals related to particular temporal fluctuations of principal cell firing. Further, CA1 principal cells reliably locked their discharge to the troughs of tSC signals, which were themselves locked to different theta phases. Moreover, we found that the different tSCs are associated with distinguishable neuronal ensembles. Taken together, these findings suggest that the cycle-to-cycle variability of tSCs reflects the functional partitioning of theta activity in distinct network operations.

### Spectral Signatures of Theta Cycles as Readouts of Distinct CA1 Operations

The synaptic currents flowing into the CA1 circuit are reflected in LFPs (Buzsáki et al., 2012). The single-cycle spectral signatures we identified here exhibit frequency signals consistent with oscillations reported previously and thought to reflect CA3 and EC3 inputs to CA1; namely, slow and mid-gamma oscillations, respectively (Bragin et al., 1995, Colgin et al., 2009, Fernández-Ruiz et al., 2017, Lasztóczi and Klausberger, 2016, Scheffer-Teixeira and Tort, 2017, Schomburg et al., 2014). In line with previous findings, we show that the mid-gamma-range tSCs extracted from CA1 pyramidal layer LFPs related to strong CSD signals in the CA1 *lacunosum moleculare* layer, whereas the currents associated with the slow gamma (tSC2) as well as the beta range (tSC1) were stronger in the CA1 *radiatum* layer. Projections of CA3 principal cells to CA1 are thought to be involved in mnemonic information retrieval, whereas ongoing information about the external world would be directly transmitted to CA1 by EC3 inputs for encoding (Hasselmo et al., 2002). Slow and mid-gamma oscillations could thus be proxies for memory retrieval and encoding (Carr and Frank, 2012), respectively. Such a suggestion is supported by recent work focusing on how gamma oscillations correlate with spatial coding schemes in CA1 (Bieri et al., 2014, Cabral et al., 2014, Dvorak et al., 2018, Zheng et al., 2016a). For example, it has been reported that slow (CA3-related) and mid (EC3-related) gamma oscillations bias CA1 spatial coding to a prospective mode (as reflected in animal crossings in which place cells tend to present a maximum instantaneous rate before their “mean” place field center) and to a retrospective mode (i.e., in animal crossings with the place cell maximum instantaneous rate delayed in relation to the mean place field center), respectively (Bieri et al., 2014). Further, CA1 place cells better represent current spatial position in theta cycles dominated by mid-gamma oscillations (Dvorak et al., 2018, Zheng et al., 2016a). Interestingly, place cell activity in theta cycles with slow gamma oscillations dominating over mid gamma oscillations better represent distant shock zones than the current location in an aversive spatial memory task (Dvorak et al., 2018). Taken together, these observations reinforce the idea of slow- and mid-gamma oscillations being more strongly associated with the retrieval of past experience and the representation of ongoing sensory information (Colgin, 2015b, Fries, 2009), respectively. This idea is also supported by recent reports of novelty-related strengthening of CA1 mid-gamma power (Bieri et al., 2014, Zheng et al., 2016b). Following this line of thoughts, cell ensembles expressed in theta cycles nesting a signal related to ongoing external information would be selectively channeled toward further processing, possibly for the purpose of consolidation. Indeed, we found that, during novel environment exploration, the principal cell co-activations in theta cycles carrying strong mid-gamma oscillations were more reactivated in SWR events of subsequent rest periods. Thus, the presence of strong tSC3 and tSC4 seems to report a CA1 operating mode during which the augmented influence of EC3 inputs, through *lacunosum moleculare* currents, conveys highly processed sensory information that drives the expression of neuronal representations associated with novel or salient behavioral experience, which would undergo enhanced offline consolidation.

We further evaluated whether tSCs were modulated during learning and probe stages of a memory task. We found that the strength of the theta-nested 80-Hz mid-gamma component (tSC4) substantially increased during learning, whereas it decreased during memory testing; the 54-Hz mid-gamma component (tSC3) instead exhibited the highest increase during memory testing. Both the beta-range (tSC1) and the slow gamma (tSC2) components also increased during the probe stage. During spatial learning, the 80-Hz mid-gamma component possibly dominates CA1 theta because new external information is paramount during this stage. Later, in the probe test, previously learned information would be retrieved from CA3 and integrated with the ongoing external information conveyed by EC3. We suggest that this is why, besides the stronger beta-range and slow gamma-range components related to *radiatum* inputs, the 54-Hz mid-gamma component related to *lacunosum moleculare* inputs is also enhanced during the memory probe test.

The strengthening of tSC4 observed during learning in the crossword maze is consistent with the previously suggested role of EC-related, ∼60- to 100-Hz gamma oscillations in memory acquisition; likewise, the probe-related increase in tSC2 is consistent with the hypothesis of a role of CA3-driven gamma oscillations in memory retrieval (Colgin, 2015b). Interestingly, we also found that the good behavioral performance observed in late learning trials was associated with stronger tSC4 and tSC2 compared with early trials. These learning-related tSC4 and tSC2 enhancements were also more pronounced near the goal location. During memory testing, tSC1 and tSC2 drastically increased near the goal and the departure zones, respectively. Importantly, such complex location-related changes in tSC strengths in the crossword maze were not observed during the baseline session, before animals experienced the behavioral relevance of these zones. These findings suggest that the strength of the theta-nested inputs received by CA1 principal cells is weighted according to stages of mnemonic information processing and behavioral relevance of spatial locations.

### Dynamic Weighting of CA1 Operations during Theta Oscillations

In this work, we found that theta cycles could be clearly dominated by a given tSC, but we did not find any evidence that the strength distributions of tSCs unveil clusters of theta cycles. Instead, we found that the different gamma oscillations and the beta signal occurred with varying levels of expression across theta cycles, indicating that tSCs could be better understood as lying along a continuous, multidimensional spectrum instead of forming discrete subsets (Figure S4). Thus, cycles dominated by a tSC would be those at the extreme of a continuum rather than representing a well-defined cluster of theta cycles. The study of theta cycles positioned at the extreme of such distributions appears to be highly valuable for the understanding of CA1 network dynamics, whereas the observations mentioned above also highlight the importance of developing theories accounting for the continuous aspect of the data. For instance, we observed that the theta cycles at the extreme of the axes defined by tSCs held distinct spike temporal patterns that represented distinct deviations from the canonical pattern derived from the grand average analysis of theta cycles. Importantly, the magnitude of such selective spike modulation diminished as the strength of tSCs also decreased in theta cycles. Moreover, we noticed that theta cycles could express multiple tSCs, although rarely, and that the strength of each tSC signal exhibited a sharply decaying autocorrelation across theta cycles. Taking these into consideration, we propose that the inputs converging to hippocampal CA1, in conjunction with local computations, are dynamically weighted and combined within each theta cycle. This opens the view that CA1 theta cycles can hold a collection of operating modes ranging from the defined network states of mainly reading (retrieving) to mainly writing (encoding).

An attractive feature of a single-mode network state view, however, is that CA1 could efficiently switch between encoding and retrieval, thereby avoiding information interference (De Almeida et al., 2012). Theoretical and experimental work also indicate that theta oscillations comprise temporally separated encoding and retrieval phases (Hasselmo et al., 2002, Siegle and Wilson, 2014). Thus, we propose that the dynamic weighting view could still explain how CA1 avoids interference through the segregation of encoding and retrieval, notably by means of distinct theta phases within a single theta cycle. Under such a scenario, EC3 and CA3 inputs would drive CA1 at different theta phases and at different levels of magnitude on a cycle-by-cycle basis and according to behavioral demands.

### The Two Facets of the CA1 Mid-gamma Band

The unsupervised spectral decomposition we applied on CA1 LFPs during theta oscillations blindly retrieved two statistically independent components within the mid-gamma range. In our framework, the spectral components were automatically extracted from their cycle-to-cycle variability, and the definition of gamma bands was therefore not derived from the averaged theta cycle. The two extracted mid-gamma tSCs both presented maximum amplitude near the theta peak; their co-firing patterns were similarly reactivated in offline SWRs, and their currents were traced back to the *lacunosum moleculare* layer. However, these 54-Hz- and 80-Hz components exhibited three important differences. First, we observed that theta cycles nesting prominent 80-Hz mid-gamma component presented increased spike discharge of CA1 principal cells around theta peak, which normally corresponds to their lowest discharge probability but tightly follows EC3 principal cell highest firing (Mizuseki et al., 2009). In contrast, the temporal fluctuation of the CA1 principal cell firing rate in theta cycles nesting the 54-Hz mid-gamma component was much weaker. Second, the strength of tSC4 showed a strong speed dependency, whereas that of tSC3 was significantly lower. Finally, tSC4 was strengthened during learning, whereas tSC3 strength increased during memory testing.

One speculative explanation is that these two mid-gamma components relate to the same network oscillator but driven at different regimes, which likely depend on the task demand. That is, the theta cycle-by-cycle variability of the mid-gamma frequency would report adaptive changes of the EC3 inputs to CA1 as the animal learns or remembers. Accordingly, the EC3-to-CA1 circuitry would oscillate at a higher mid-gamma frequency while the animal is actively engaging in learning and the CA1 circuit is under a strong encoding load, pushing theta spectral signatures to the 80-Hz range and increasing its effect on principal cell firing. During periods dominated by memory retrieval, the hippocampal network would then operate in a different regime, during which *radiatum* currents conveying previously learned information are integrated with external information coming to the *lacunosum moleculare* layer. In such a case, the mid-gamma oscillations would oscillate at a lower frequency because of a milder load on the EC3-to-CA1 circuitry compared with the learning stage. During memory retrieval, the milder influence of tSC3-related currents on the CA1 principal cell firing rate (compared with tSC4) could avoid interference with *radiatum* layer-driven currents, allowing the reliable reinstatement of firing patterns associated with CA3 inputs.

An alternative possibility is that tSC3 and tSC4 relate to two different oscillators. Although the CSD analysis indicates that both tSC3 and tSC4 reflect currents in the *lacunosum moleculare* layer, these two mid-gamma tSCs could be the readouts of different EC populations projecting to CA1. Our results show that tSC4 is more speed-modulated than tSC3 and that tSC4 also correlates more positively with speed-modulated principal cells. This raises the possibility that tSC4-related oscillations reflect the activity of a subset of EC cells with strong speed modulation, which have been reported previously (Kropff et al., 2015, Sun et al., 2015), whereas tSC3-related oscillations would reflect EC cells with weak speed correlation.

Overall, these findings support the notion that individual theta cycles represent versatile temporal units in which CA1 computations are tuned to transiently shape principal cell firing output during spatial exploration and memory-guided behavior. These findings also highlight the importance of single-cycle analysis of theta oscillations in deciphering fine-grained CA1 circuit dynamics.

## STAR★Methods

### Key Resources Table

**Table:** 

REAGENT or RESOURCE	SOURCE	IDENTIFIER
**Experimental Models: Organisms/Strains**		

CamKIIa-Cre B6.Cg-Tg(Camk2a-cre)T29-1Stl/J mice	https://www.jax.org	stock number 005359; RRID: IMSR_JAX:005359
Dat-Cre B6.SJL-Slc6a3tm1.1(cre)Bkmn/J mice	https://www.jax.org	stock number 006660; RRID: IMSR_JAX:006660
Long Evans rats	Harlan, UK	Strain code 140
C57BL/6J mice	Charles River, UK	Strain code 632

**Software and Algorithms**		

Intan RHD2000	Intan Technologies, Los Angeles	http://intantech.com/RHD2000_evaluation_system.html
KlustaKwik	Harris et al., 2000, Kadir et al., 2014	https://github.com/klusta-team/klustakwik/
Python 2.7	https://www.python.org	Python 2.7.13
PTSA package	https://pennmem.github.io/ptsa_new/html/index.html	v2.0.3
Scikit-learn 0.18.1 (Python package)	http://scikit-learn.org/stable/	v0.18.1
Stats model (Python package)	http://www.statsmodels.org/stable/index.html	v0.8.0
Theta Spectral Component Extraction	https://data.mrc.ox.ac.uk/data-set/tsc	v1.0

**Other**		

12um tungsten wires	California Fine Wire	M294520
Silicon probe	Neuronexus	A1x32-6mm-50-177-H32_21mm
64-channels amplifier	Sensorium Inc., Charlotte, VT	EPA-6
Head-stage amplifier	Intan Technologies, Los Angeles	RHD2164

### Experimental Model and Subject Details

Animals used were male adult (4–7 months old) C57BL/6J mice (Charles River, UK) or transgenic heterozygous Cre-driver mice (Jackson Laboratories; obtained from C57BL/6J crossed with CamKIIa-Cre B6.Cg-Tg(Camk2a-cre)T29-1Stl/J, stock number 005359, RRID: IMSR_JAX:005359; or Dat-Cre B6.SJL-Slc6a3^tm1.1(cre)Bkmn^/J, stock number 006660, RRID: IMSR_JAX:006660). In addition, we used adult male Long-Evans rats (Harlan, UK) to test rat dorsal hippocampal CA1 LFPs for the presence of the theta-nested spectral components we identified in mice. All animals had free access to water and food in a dedicated housing facility with a 12/12 h light/dark cycle. They shared a cage with their littermates until the surgery. All experiments involving animals were conducted according to the UK Animals (Scientific Procedures) Act 1986 under personal and project licenses issued by the Home Office following ethical review.

### Method Details

#### Microdrive implantation

Animals were implanted with a custom-made microdrive during a surgical procedure performed under deep anesthesia using isoflurane (0.5%–2%) and oxygen (2 l/min), with analgesia (0.1 mg/kg vetergesic) provided before and after. The drive was designed with tetrodes (10–12 in mice; 16 in rats) aimed at the stratum pyramidale of the dorsal CA1 hippocampus (Dupret et al., 2010, van de Ven et al., 2016). Tetrodes were constructed by twisting together four insulated tungsten wires (12 μm diameter, California Fine Wire) and shortly heating them to bind them together in a single bundle. Each tetrode was attached to a M1.0 screw to enable their independent movement. The drive was implanted under stereotaxic control in reference to bregma (Dupret et al., 2010, van de Ven et al., 2016). Tetrodes were initially implanted above the CA1 pyramidal layer and their exposed parts were covered with paraffin wax. The drive was then secured to the skull using dental cement. For extra stability, stainless-steel anchor screws had first been inserted into the skull. Two of the anchor screws, which were inserted above the cerebellum, were attached to 50 μm tungsten wires (California Fine Wire) and served as ground and reference electrodes during the recordings. The placement of the tetrodes in dorsal CA1 was confirmed by the electrophysiological profile of the local field potentials in the hippocampal ripple frequency band and anatomical electrode tracks, as previously described (Csicsvari et al., 1999, van de Ven et al., 2016). In one additional mouse, a single-shank silicon probe (Neuronexus, model A1x32-6mm-50-177-H32_21mm) was implanted following the same surgical procedure to assess the CA1 laminar profile of the theta-nested spectral components (Figure 5).

#### Recording procedures

Recordings commenced following full recovery from the surgery. For the recordings involving spatial exploration of open-field enclosures, each animal was connected to the recording apparatus and familiarized with a high-walled box containing home cage bedding (the “sleep-box”) and with one of the open-field enclosures (the familiar enclosure) over a period of approximately seven days. During this period, tetrodes were gradually lowered to the stratum oriens of the hippocampal CA1. On the morning of each recording day, tetrodes were further lowered into the pyramidal cell layer in search of multi-unit spiking activity and sharp-wave/ripple events (Dupret et al., 2010, van de Ven et al., 2016). Tetrodes were not moved for at least 1.5 h before recordings started. For each recording day, the animal was first recorded in the sleep-box (“pre-exploration rest,” ∼25 min). The animal was then recorded during an open-field exploration session (∼25 min) followed by another sleep-box session (“post-exploration rest”). The open-field enclosure was either the familiar enclosure, which the animal had repeatedly been exposed to before, or a novel enclosure the animal had never seen before. The open-field enclosures differed in shape and in the cue-cards that lined some of the walls. The results on open-field experiments reported in the present study are based on a total of 20 mouse tetrode recording days (including 18 familiar enclosure and 12 novel enclosure sessions, with mice exposed both to a familiar and a novel enclosure in most of the recording days; some of these recordings were used as control recording days in a previous study by van de Ven et al. (2016)), 8 rat tetrode recording days and 4 mouse silicon probe recording days.

To assess the functional significance of theta-nested spectral components in memory-guided behavior, additional mouse recordings were performed on the crossword maze. Some of these recordings were used as control recording days in a previous study by McNamara et al. (2014). The crossword-like apparatus consisted of four departure boxes and eight intersecting open tracks forming fourteen intersections inspired by a layout used in the seminal study of Tolman and Honzik (1930). The width of each track was 5 cm with a 1.5 cm high rim along the edges. The entire maze measured 95 cm^2^ excluding start boxes. The maze was painted black and suspended 5 cm above a black table. Distal cue cards were placed on the curtain surrounding the maze and some cue objects were placed on the supporting table dispersed throughout the maze. In order to promote spatial navigation by distal cues, the maze was randomly rotated relative to the cues at the beginning of each day. Mice performing the crossword maze task were maintained at 85% of their post-operative body weight. On each day, the animal was allowed to explore the maze with the departure boxes closed and in the absence of intra-maze barriers and rewards for approximately 20 min (baseline session). For the learning stage, two departure boxes and one food reward location (at the end of one of the five tracks protruding from the maze) were selected as in use for that day and the maze was configured with a new arrangement of up to seven barriers (10 cm in height) such that there was only one path from each departure box to the reward. Mice were given up to 20 trials to learn to find the reward with the start point randomly switching between the two departure boxes. The per trial reward was 4 μl of condensed milk diluted 30% in water and was placed on a plastic cap at the goal location. A similar plastic cap (without reward) was placed in each of the other 4 tracks protruding from the maze. A glass vial (with perforated lid) containing an aliquot of the reward yet non accessible was placed inside the two departure boxes to signal the onset of the learning stage to the animal. The board was cleaned after each learning trial to prevent the use of an odor guided search strategy. The memory probe test was conducted 1 hour after learning with the maze maintained in the same layout as the learning stage but without any reward. The present study includes a total of 13 recording days on the crossword maze.

At the end of each recording day, tetrodes were raised to the stratum oriens to avoid damaging the pyramidal layer overnight.

#### Multichannel data acquisition and position tracking

The extracellular signals from the electrodes were buffered on the head of the animal (unity gain op-amps, Axona Ltd) and transmitted over a single strand of litz wire to a dual stage amplifier and band pass filter (gain 1000, pass band 0.1 Hz to 5 kHz; Sensorium Inc., Charlotte, VT), or (in other setups) the electrode signals were amplified, multiplexed, and digitized using a single integrated circuit located on the head of the animal (RHD2164, Intan Technologies, Los Angeles; pass band 0.09 Hz to 7.60 kHz). The amplified and filtered electrophysiological signals were digitized at 20 kHz and saved to disk along with the synchronization signals from the position tracking. LFPs were further down-sampled to 1250 Hz for all subsequent analyses. In order to track the location of the animal three LED clusters were attached to the electrode casing and captured at 39 frames per second by an overhead color camera.

#### Spike detection and unit isolation

For the offline detection of spikes, the recorded signals were first band-pass filtered (800 Hz to 5 kHz). Spikes were then detected based on the power (root-mean-square) of the filtered signal calculated in 0.2 ms sliding windows. Detected spikes of the individual electrodes were combined per tetrode. To isolate spikes belonging to the same neuron, spike waveforms were first up-sampled to 40 kHz and aligned to their maximal trough (Csicsvari et al., 1998). Principal component analysis was applied to these waveforms ± 0.5 ms from the trough to extract the first three or four principal components per channel, such that each individual spike was represented by 12 waveform parameters. An automatic clustering program (KlustaKwik, http://klusta-team.github.io) was run on this principal component space and the resulting clusters were manually recombined and further isolated based on cloud shape in the principal component space, cross-channels spike waveforms, auto-correlation histograms and cross-correlation histograms (Harris et al., 2000, Kadir et al., 2014). All sessions recorded on the same day were concatenated and clustered together. Each cluster used for further analysis showed throughout the entire recording day stable cross-channels spike waveforms, a clear refractory period in its auto-correlation histogram, well-defined cluster boundaries and an absence of refractory period in its cross-correlation histograms with the other clusters. Hippocampal principal neurons were identified by the shape of their auto-correlation histogram, their firing rate and their spike waveform (Csicsvari et al., 1998).

### Quantification and Statistical Analysis

Data were analyzed in Python 2.7 (https://www.python.org/downloads/release/python-2714/) using the python packages mentioned below.

#### Unsupervised decomposition of local field potentials (LFPs)

We applied Ensemble Empirical Mode Decomposition (EEMD) to extract low-frequency, theta and supra-theta signals from raw LFPs (Figure S1) using the PSTA package (https://pennmem.github.io/ptsa_new/html/index.html). The EEMD consists of breaking down a time varying, non-stationary signal into its elementary signals referred to as the Intrinsic Mode Functions (IMFs) by iteratively applying the empirical mode decomposition algorithm with added white noise to prevent mode mixing (Wu and Huang, 2009). We extracted the theta signal of each raw LFP by combining the IMFs with mean instantaneous frequencies between 5 and 12 Hz. Low-frequency and supra-theta signals were defined as the sum of IMFs with mean frequencies below 5 Hz and above 12 Hz, respectively (Figure S1). Note that we used EEMD to obtain the theta waveform and avoid harmonic artifacts related to cycle asymmetries. Besides being an unsupervised filter (i.e., free of predefined frequency bands), one of the main advantage of the EEMD is that it deals well with asymmetrical (non-linear) and non-stationary signals, thus diminishing filtering artifacts (such as harmonics and side band-related distortions) caused by convolution filters for cross-frequency coupling analysis (Aru et al., 2015, Belluscio et al., 2012, Yeh et al., 2016). Therefore, apart from having the theta signal automatically extracted from the raw LFP, the EEMD also provides supra-theta components that are virtually free from harmonic artifacts (Wu and Huang, 2009).

#### Extracting and quantifying theta spectral components (tSCs)

To identify individual theta cycles we detected candidate peaks and troughs (local maxima and minima of the theta signal from the EEMD) during periods of active exploratory behavior (animal speed > 2 cm/s) and with absolute values above the envelope of the low-frequency signal. A theta cycle was defined by each pair of consecutive candidate troughs separated at least by 71ms (∼14 Hz) and no more than 200ms (5 Hz) that surrounded a candidate peak. We next averaged the spectrogram of the supra-theta signal within each detected theta cycle (from trough to trough). Spectrograms were computed with a set of complex Morlet wavelets with main frequencies from 10 to 200 Hz, 1-Hz steps (using scipy.signal.morlet function). The spectral signature of each theta cycle was defined as the obtained vector carrying the mean amplitude of each spectrogram frequency. We applied Independent Component Analysis (ICA) to the set of extracted spectral signatures using the FastICA algorithm from the scikit-learn package (http://scikit-learn.org/stable/). Prior to ICA, dimensionality reduction of the data was performed by principal component analysis and we used the first 5 components, which accounted for 85% of the variance. We defined the theta-nested Spectral Components (tSCs) as the extracted independent components. Note that this approach does not require *a priori* knowledge of the frequency bands defining each oscillation, as ICA allows extracting, in an unsupervised manner, statistically independent mixtures of supra-theta frequency components in a cycle-by-cycle basis.

The distribution of the projection of a given tSC onto the set of spectral signatures was typically asymmetrical, akin to a Gaussian-like distribution with one long tail (e.g., Figure S4B). The sign of each tSC vector was defined as the one that made its mean projection onto the data (inner product between the tSC and all spectral signatures) positive (Figures 1C, S2A, S2D, S2H, and S8B). We defined as the strength of a given tSC, the projection of that tSC onto a single cycle spectral signature (e.g., Figure 3A) or onto a time point of the spectrogram (Figure 2A). As tSCs were coherent across all CA1 pyramidal layer tetrodes (inter-tetrode Pearson correlations of tSC strength: tSC1 = 0.65 ± 0.11; tSC2 = 0.64 ± 0.12; tSC3 = 0.69 ± 0.11; tSC4 = 0.74 ± 0.11; mean across averaged inter-tetrode pairwise correlations of all mouse recording days ± standard deviation), the tetrode with the highest gamma power was used for subsequent analyses.

For analyses evaluating theta cycles nesting strong tSC signals (Figures 1, 2, 5, and S3–S5) we used a threshold for the distribution of the single cycle tSC strengths, as:

(Equation 1) $$Threshold=\frac{2\times median\left(\left|p-median\left(p\right)\right|\right)}{0.6745}+median\left(p\right)$$

Where p is the distribution of strengths of a given tSC, and median(|p|)/0.6745 is the estimation of the standard deviation of p not considering outlying values (Donoho and Johnstone, 1994).

To calculate the theta phase coupling of tSCs strength, theta phases were computed by linearly interpolating values between troughs, zero-crossings and peaks (Belluscio et al., 2012). The strength of tSCs as a function of theta phase (Figures 2A and S3C) was calculated as the inner product between a given tSC and each time point of the supra-theta spectrograms (computed as before, namely with a set of complex Morlet wavelets with main frequencies from 10 to 200 Hz, 1-Hz steps using the scipy.signal.morlet function). This allowed obtaining the time course of the tSC at the same temporal resolution as the LFPs (i.e., 1250 Hz). The tSC strength was then z-scored and correlated with ongoing theta phase.

#### Principal cell spiking activity in tSC cycles

The firing activity of principal cells was triggered to the peaks of the theta cycles of each tSC or to the peaks of all theta cycles (Figures 2B and S3D) using 0.8ms time bins in order to match the time resolution of the LFPs. For display purpose, the triggered averages were smoothed with a Gaussian kernel (10 ms standard deviation). Z-scores were computed using all theta cycles. When comparing the instantaneous firing rate of principal cells between tSCs around theta peaks (Figure 2B), we evaluated the mean rate (z-scored spike count) of principal cells within a 20-ms window centered at the peaks of cycles assigned to each tSC using Wilcoxon signed-rank tests. The same procedure was used to test for differences in principal cell firing rate around theta troughs.

To evaluate the changes in spike probability of principal cells (SPCs) as a function of ongoing theta phase (Figure 2C) we first calculated the mean rate of each neuron for a given theta phase. The spike probability change (%) for a given theta phase and tSC was then calculated as:

(Equation 2) $$SPC\left(\varphi \right)=100\times \frac{rate_{tSC}\left(\varphi \right)-rate_{overall}\left(\varphi \right)}{rate_{overall}\left(\varphi \right)}$$

Where $rate_{tSC}\left(\varphi \right)$ and $rate_{overall}\left(\varphi \right)$ are the mean rates of the neuron for theta phase φ computed from the theta cycles strongly nesting a given tSC or for all theta cycles, respectively. SPCs were computed for all principal cells individually and then averaged. Theta phases were divided in 60 equally spaced bins and SPCs were circularly smoothed with a Gaussian kernel (24 degree standard deviation). For this analysis, we defined the bounds of each cycle as the theta signal ascending zero-crossings surrounding the cycle peak (i.e., each cycle was bounded from the zero-crossing preceding its peak to the zero-crossing immediately following its trough). Note that although tSCs’ amplitude is maximal at different theta phases, none of them, nor the principal cell rate, peak along the CA1 pyramidal layer theta ascending phase. This was done for spike analysis in order to avoid cutting the theta cycle at its (second) trough where principal cell firing rate is maximal. The same quantification was used for evaluating SPCs in theta cycles of different tSC slices shown in Figures S5B and S5C.

The statistical significance of SPC(φ) values was established using an ANOVA model:

(Equation 3) $$SPC_{tSC_{i},n}\left(\varphi \right)=\beta_{0}+\sum_{tSC_{i}=1}^{5}\beta_{tSC_{i}}T_{tSC_{i}}+\sum_{mouse_{id}}\beta_{mouse_{id}}M_{mouse_{id}}+error_{tSC_{i},n}$$

Where $SPC_{tSC_{i},n}\left(\varphi \right)\;$ is the mean spike probability change of neuron n for theta cycles assigned to tSCi for theta phaseφ; $T_{tSC_{i}}$ is the categorical variable receiving value 1 to designate that the observation came from $tSC_{i}$ theta cycles and 0 otherwise. Likewise, $M_{mouse_{id}}$ is the categorical variable referring to animal identity. The term $\sum \beta_{mouse_{id}}M_{mouse_{id}}$ was used to control for inter-mouse variance. A recorded mouse was randomly selected as reference (treatment) to avoid collinearity issues, known as the dummy variable trap (Gujarati and Porter, 2009). However note that this does not affect $\beta_{tSC_{i}}$. We determined rate increases or decreases as significant when $\beta_{tSC_{i}}$ coefficients presented p values below (0.05/((5tSCs)×(60thetaphases))≈0.00017) in at least 5 consecutive theta phases. The ANOVA model was fitted through the statsmodels.formula.api Python module (http://www.statsmodels.org/stable/index.html).

#### Phase coupling of principal cell spiking to tSC signals

We defined each tSC signal as the IMF with the closest main frequency to a given tSC peak frequency. For spike to tSC phase analyses, the instantaneous phases of tSC signals were computed through the Hilbert Transform. Then, the tSC phases were sampled by spikes of a given neuron happening within theta cycles of the corresponding tSC or within theta cycles in different tSC strength slices (Figures 2E, S5D and S5E). The spike-phase coherence was quantified as the mean vector length of such distribution of phases (Siapas et al., 2005). The distributions of the mean firing phase of principal cell spikes to tSC signals (Figures 2E, S3E, and S5D) only included neurons with spike-phase coherence higher than the 99.9^th^ percentile of their corresponding control distribution (p < 0.001). Control distributions were computed by randomly circularly shifting principal cell spikes to different theta cycles while preserving their original theta phase. The theta phase distribution of the neuron was therefore not changed in such controls, and the spike-phase coherence to the tSC signal was recomputed. Each control distribution consisted of 2000 of such shifted coherences. Further, in order to avoid spike waveform contamination biases, tSC phases were never taken from the electrode the neuron was recorded from. Proportion of cells significantly coupled: tSC1: 82.9%, tSC2: 65.4%, tSC3: 36.5%, tSC4: 33.2%. The same procedure was repeated for theta cycles nesting multiple tSCs by taking only cycles with strengths of a pair of tSCs above their threshold. Only neurons with a total of > 100 spikes were considered.

The spike timing of principal cells triggered to each tSC signal (Figures 2D, S3F and S3H) was assessed by first detecting the troughs of that tSC signal within its corresponding theta cycles. Only the most negative trough within each theta cycle was used. A given theta cycle never contributed with more than one tSC signal trough to prevent tSC signal auto-correlations to bias triggered averages at short temporal scales (intra-theta cycle). The tSC signal was only regarded within the bounds determined by theta cycles assigned to the corresponding tSC.

#### GLM prediction of tSC strength from principal cell activity

Each GLM was fitted to predict tSC strength in all individual theta cycles from the activity of simultaneously recorded principal cells using scikit-learn. As before, the activity of a principal cell in a given theta cycle was quantified by its spike count between the theta signal ascending zero-crossings immediately before and after the cycle peak (see Principal Cell Spiking Activity in tSC Cycles section above). In sum, the principal cell activity was represented by a matrix, in which columns represent theta cycles and rows represent single neurons. All GLMs were 10-fold cross-validated. The set of cycles used for fitting the model are referred to as the training set, whereas the cycles with tSC strength being predicted are referred to as the testing set. This process was repeated until all groups were used as testing sets (i.e., by the end of the cross-validation, the GLM predicted strength of each tSC in all recorded theta cycles). The activity of principal cells were z-scored prior to the prediction, and such standardization was also cross-validated. More specifically, the mean and standard deviation of the spike counts used for z-scoring were computed only from the training set and then applied to all cycles. Same cross-validated standardization was used for tSC strengths. Once the tSC strengths of all cycles were predicted, the accuracy of the prediction was quantified as the Pearson correlation between the actual and the predicted values. In order to test the significance of such predictions, we repeated this procedure after shifting spike trains across theta cycles. More specifically, the columns of the spike count matrix of principal cells were circularly shifted. Importantly, all columns were shifted together, so the correlation between neurons was preserved as well as the autocorrelation of each individual neuron. However, the original relationship between the spike counts and the tSC strength was destroyed. For each recording session, we computed 1000 of such prediction shift controls. Original predictions higher than the 99^th^ percentile (p value < 0.01) of the control distribution were regarded as significant. Proportion of recording sessions with significant tSC predictions across the whole dataset: tSC1: 82.4%; tSC2: 64.7%; tSC3: 56.9%; tSC4: 94.1%; all p < 0.01; shift prediction tests.

In order to compare between predictions achieved when the training and testing sets were taken for the same tSC to the ones obtained for mismatched sets (Figure 3C, left), we standardized predictions to their corresponding control distributions, as follows:

(Equation 4) $$\hat{r}=\frac{r-mean\left(r_{control}\right)}{std\left(r_{control}\right)}$$

Where r is the actual prediction, and $mean\left(r_{control}\right)$ and $std\left(r_{control}\right)$ are the mean and standard deviation of the control distribution, respectively. We also normalized predictions by dividing them by the ones obtained when the training and testing sets were taken from the same tSC (Figure 3C, right).

We also evaluated the relationships between tSC GLM weights, speed modulation and spatial information of individual neurons. Speed modulation was quantified as in Figures S2F and S2G but for individual neurons by using their spike counts across theta cycles (instead of the tSC strength used in Figures S2F and S2G). Spatial information was computed as in Markus et al., (1995). More specifically, the spatial information of a given neuron was defined as:

(Equation 5) $$\sum_{i}P_{i}\left(R_{i}/R\right)\log_{2}\left(R_{i}/R\right)$$

where $P_{i}$ and $R_{i}$ are the probability of occupancy of spatial bin *i* and the mean firing rate of that neuron in spatial bin *i*, respectively; and R is the overall mean rate of that neuron. Then, in order to test if the speed modulation of individual neurons was related to a given tSC GLM, we computed the Spearman correlation between the weights of each GLM and the speed modulation (Figure S6A) or spatial information (Figure S6B). Thus, a Spearman correlation between tSC GLM weight and speed modulation (or spatial information) was obtained for each session. Then, we compared Spearman correlation values obtained for different tSCs (Figures S6A and S6B, right panels).

Finally, we performed two control analyses to test whether the relations obtained from the GLMs between tSC strength and neuronal firing (Figure 3) could be explained by their co-modulation by speed. In the first analysis, we re-computed cross-validated GLMs as before, but only using theta cycles with speed values within a 2-cm/s instantaneous speed bin (1–3cm/s, 3–5cm/s 5–7cm/s, 7-9cm/s and 9-11cm/s). Thus, we obtained for each GLM a prediction score for each of these speed bins and their corresponding shuffling controls (averaged across 1000 circular shift controls). Then, prediction of different speed bins were averaged for each tSC in each session and were compared to their corresponding controls (see corresponding Results section of main text). In the second analysis (Figure S6C), we tested whether spike trains would provide additional information to GLMs trained to predict tSC strength from speed. The underpinning rationale was that if the prediction power of spike trains was solely explained by speed, then including spike information to a model that already contained speed as a regressor would not increase that model’s performance. We recomputed the cross-validated GLMs and their prediction performance in three conditions where the data from each theta cycles corresponded to either: (1) the original speed with shuffled spikes (i.e., spikes coming from another cycle using a random circular shift of spike counts across theta cycles), (2) the original spikes with shuffled speed (i.e., same as before but with circular shift of speed values across theta cycles), or (3) the original speed with original spikes. The prediction for conditions 1 and 2 were taken as the mean of 1000 realizations. We compared across GLMs with same number of regressors in order to account for biases due to the complexity of the model and due to autocorrelation of the shuffled features.

#### SWR reactivation

To detect SWR events, LFPs were referenced to a ripple-free electrode and band-pass filtered (135-250 Hz). The power (root mean square) of the filtered signals were then calculated and summed to reduce variability. The threshold for SWR detecting was set as 7 standard deviations from the mean power (Csicsvari et al., 1998, van de Ven et al., 2016). Only SWR events happening within periods where the instantaneous speed of the animal was less than 2cm/s were considered. For familiar versus novel reactivation analysis, we ensured that each exploration session contributed with the same number of spectral signatures (determined by the session with the least number of theta cycles) for tSC extraction to avoid biases due to possible unbalanced number of theta cycles across recordings sessions. To calculate theta co-firing (Figure 4), we calculated the spike counts of each principal cell during exploration in theta windows defined by the ascending zero-crossings immediately before and after theta peaks and then we calculated the (Pearson) correlation coefficient between each cell pair using theta cycles with the strongest strength of a given tSC. Likewise, SWR co-firing values were calculated as the correlation coefficients between the spike counts of principal cell pairs taken from SWR windows (100ms windows centered on the supra-threshold peaks of the ripple power).

The SWR reactivation strength $\beta_{tSC_{i}}$ for $tSC_{i}$ theta cycles (Figure 4B; Theta co-firing versus SWR co-firing) was defined by the following regression (using statsmodels.formula.api):

(Equation 6) $$SWR_{post,p}=\beta_{0}+\beta_{tSC_{i}}\theta_{tSC_{i},p}+\beta_{SWR_{pre}}SWR_{pre,p}+error_{p}$$

Where $SWR_{post,p}$ and $SWR_{pre,p}$ are the SWR co-firing of the pair of neurons p during pre- and post-exploration sleep/rest epochs, respectively; $\theta_{tSC_{i},p}$ is the theta co-firing of p in $tSC_{i}$ theta cycles. The term $\beta_{SWR_{pre}}SWR_{pre,p}$ was included to control for the correlation structure present in pre exploration SWRs.

In a complementary analysis, we fitted a linear regression to predict the change in SWR co-firing (from pre-exploration rest to post-exploration rest) from theta co-firing (Figure 4C), as follows:

(Equation 7) $$\;\Delta SWR_{p}=SWR_{post,p}-SWR_{pre,p}=\beta_{0}+\sum_{i}\beta_{tSC_{i}}\theta_{tSC_{i},p}+error_{p}$$

Therefore, the contribution of each tSC to $\Delta SWR_{p}$ are estimated by its corresponding $\;\beta_{tSC_{i}}$.

In order to evaluate if speed modulation of tSC strength could explain SWR reactivation results, we also performed a speed- and location-matching control for familiar/novel enclosures (Figure S7). We implemented such a control analysis because, for example, the SWR reactivation enhancement observed for tSC4 following exploration of novel environments could come from cycles with high speeds, since tSC4 is highly and positively correlated with speed. Each control was computed by replacing each original theta cycle of a given tSC by another random cycle with similar speed (no more than 0.5 cm/s difference) and occurring at a nearby location (no more than 5 cm away; when no speed-matched cycle was available within that maximum distance, the closest cycle within the same speed bin was selected). In this way, each control was composed by a set of theta cycles with virtually the same speed and spatial distributions (Figure S7B) as the original set of theta cycles. For each tSC and condition (familiar or novel), we ran 1000 of such controls. Finally, we compared SWR reactivation obtained as before (Figure 4B) to theta cycles matched by speed and location (Figure S7C).

#### Current source density analysis

Current sources and sinks were estimated from LFP recordings from a silicon probe implanted through the somato-dendritic axis of CA1. The current source density (unscaled) signal at time *t* and electrode *n*, $CSD{\left[t\right]}_{n}$, was estimated as (Brankack et al., 1993, Lasztóczi and Klausberger, 2014, Mitzdorf, 1985):

(Equation 8) $$CSD{\left[t\right]}_{n}=-\left(LFP{\left[t\right]}_{n-1}-2*LFP{\left[t\right]}_{n}-LFP{\left[t\right]}_{n+1}\right)$$

Where $\;LFP{\left[t\right]}_{n-1}$, $LFP{\left[t\right]}_{n}$ and $\;LFP{\left[t\right]}_{n+1}$ are the LFP signals at time *t* recorded from neighboring electrodes (50 μm apart). The silicon probe recording site in the pyramidal layer was identified as the one with largest ripple-band power. We defined the location of radiatum and lacunosum moleculare layers according to the ripple and sharp-wave laminar profiles and electrode spacing. The amplitude of different frequencies of CSD signals were computed by the same wavelet framework we used for LFPs before (e.g., Figure 1D) similarly to (Lasztóczi and Klausberger, 2014, Lasztóczi and Klausberger, 2016, Lasztóczi and Klausberger, 2017). Note that with this approach, CSDs were not computed from filtered LFPs nor from averaged signals of a chosen reference (i.e., through of a given oscillation at a particular channel), but from “raw CSD signal” time courses. Importantly, tSCs were always identified from the pyramidal layer LFP (Figure S2H), following the procedure used in tetrode recordings. Likewise, tSC cycles (Figure 5C) were defined from pyramidal layer LFPs, as in Figure 1D.

#### Analysis of tSC strength modulation by task stages and spatial location on the crossword maze

tSCs were first extracted (as before) using the theta cycles recorded in the baseline session, and then their strength was computed in all task stages (Figure 6A). Throughout this analysis, the tSC strengths were z-scored relative to baseline to evaluate their changes during task stages. Thus, the normalized strength of a tSC in a given theta cycle was defined as:

(Equation 9) $$tSC\;str_{Zbaseline}=\frac{tSCstrength-\;mean\left(tSC_{strength}^{baseline}\right)}{\;std\left(tSC_{strength}^{baseline}\right)}$$

Where $tSC_{str}$ is the tSC strength in that cycle and $mean\left(tSC_{strength}^{baseline}\right)$ and $std\left(tSC_{strength}^{baseline}\right)$ are the mean and standard deviation of the tSC strength in baseline.

We quantified the modulation of a task stage (learning or probe) on the tSC strength according to the following ANOVA regression model:

(Equation 10) $$tSC\;str_{Zbaseline}=\beta_{0}+\beta_{stage_{id}}S_{stage_{id}}+\beta_{speed}Speed+\sum_{recday_{id}}\beta_{recday_{id}}R_{recday_{id}}$$

Where $S_{stage_{id}}$ is the categorical variable receiving value 1 to designate that the observation came from the learning (or probe) stage and 0 if it came from baseline. Likewise, $R_{recday_{id}}$ denotes the categorical variable referring to recording day identity. Finally, Speed carries the animal’s speed on that theta cycle. Thus, the modulation of a particular stage (relative to baseline) onto the strength of a tSC is defined as $\beta_{stage_{id}}$ (Figures 6C and 6D), whereas $\beta_{speed}$ refers to the speed modulation of that particular tSC. Intuitively one can understand $\beta_{speed}$ as the slope observed when expressing tSC strength as function of speed (Figure 6B), whereas $\beta_{stage_{id}}$ captures the offset between the data from learning (or probe) stage relative to baseline (Figure 6B).

To rule out the possibility that the amount of time spent at different speeds or locations in different stages could skew the results in Figure 6, we ran an additional matching control analysis. For that, speed and location of cycles from the learning and baseline stages were matched to the probe, as the latter was always the stage with fewer theta cycles (i.e., shorter recording session). More specifically, for each theta cycle in a probe test, we randomly selected one from learning (or baseline) with similar speed and location (from no more than 5 cm away and 0.5 cm/s speed difference).

We also evaluated if the strength of different tSCs was different between the early and late learning trials. For that, we ran match controls between early and late trials by randomly selecting, for each theta cycle detected in the last three trials, one theta cycle with similar speed and location (from no more than 5 cm away and 0.5 cm/s speed difference) from the first three trials. For each control, we then averaged the normalized (as before) tSC strength in the same recording day and then averaged across recording days. Statistics were performed using these bootstrap distributions. In order to rule out the possibility that the obtained statistical differences were due to few recording days, we repeated the same procedure but removing every combination of two recording days (i.e., re-computing results for all combinations of 11 out of 13 recording days). We found that all statistical differences between early and late trials (shown in Figure S8H) held for all combinations.

To assess whether changes in the strength of tSCs could relate to behaviorally-relevant locations, we computed tSC strengths of theta cycles detected in three zones with particular interest for goal-directed behavior on the crossword maze: Departure, Barrier and Goal zones (Figure S8I). Departure and Goal zones were defined as being within 20 cm path distance to the departure boxes and the reward location, respectively. Barrier zones were defined as being 10 cm away from the intra-maze barriers (Euclidean distance). We used similar speed-matching control as for the comparison between early and late learning trials (Figure S8H) but by matching the speed of each theta cycle detected in the Departure (or Barrier) zones to a theta cycle detected in the Goal zone, as the former zone had fewer cycles; this speed-matching was performed for theta cycle detected within each task stage.

#### Analysis of amplitude and speed modulation of predefined gamma bands by task stages on the crossword maze

We repeated the same analysis shown in Figure 6, but for predefined frequency bands (Figures S8J–S8M). Individual theta cycles were defined as before. However, instead of using tSC strength as computed by ICA, we analyzed the amplitude of slow- and mid-gamma frequency bands in the detected theta cycles. Slow- and mid-gamma amplitudes were defined by (1) filtering raw LFPs by means of a Butterworth filter (2^nd^ order; 22 to 55 Hz and 60 to 100 Hz cutoff frequencies for slow- and mid-gamma, respectively); (2) calculating the instantaneous amplitude of each filtered signal with the Hilbert transform; and (3) averaging such amplitudes within the bounds of each theta cycle (defined by EEMD, Figure S1). Then, the same analyses performed for tSC strengths in Figure 6 were performed with such gamma bands.

### Data and Software Availability

The software used for data acquisition and analysis are available for download using the web links mentioned above. Data will be made available upon request.

## Supporting Citations

The following references appear in the Supplemental Information: Lex et al., 2014, Scheffer-Teixeira et al., 2013.

## Supplemental Information

Document S1. Figures S1–S8

Document S2. Article plus Supplemental Information
