# Supplementary material for: Parsing Hippocampal Theta Oscillations by Nested Spectral Components during Spatial Exploration and Memory-Guided Behavior
Source: Neuron. 2018 Nov 21;100(4):940–952.e7. doi: 10.1016/j.neuron.2018.09.031 (PMC6277817; doi:10.1016/j.neuron.2018.09.031)
Supplement: Document S1. Figures S1–S8 [file mmc1.pdf]

Neuron, Volume *100*

Supplemental Information

Parsing Hippocampal Theta Oscillations by Nested Spectral Components during Spatial Exploration and Memory-Guided Behavior

Vítor Lopes-dos-Santos, Gido M. van de Ven, Alexander Morley, Stéphanie Trouche, Natalia Campo-Urriza, and David Dupret

Supplemental Information

Parsing hippocampal theta oscillations by nested spectral components during spatial exploration and memory-guided behavior

Vítor Lopes-dos-Santos, Gido M. van de Ven, Alexander Morley, Stéphanie Trouche, Natalia Campo-Urriza and David Dupret

Inventory of Supplementary Figures

Figure S1, related to **Figure 1.**
Shows power spectra of LFP intrinsic mode functions extracted by EEMD and used to compose the theta and supra-theta signals, which are then fed into the tSC extraction framework.

Figure S2, related to **Figures 1** and **5.**
Shows the robustness of tSC extraction across recording days for both tetrode recordings in mice and rats, as well as for silicon probe recordings in mice. It also displays group data for tSC waveform, autocorrelation and speed modulation.

Figure S3, related to **Figures 1** and **2.**
Shows same analyses shown in Figures 1 and 2 but for tSC5 cycles. Additionally, it also shows that the underlying elements contributing to tSC5 strength can either be spike waveform leakage as well as fast-gamma oscillations.

Figure S4, related to **Figures 1** and **2.**
Shows raw LFP spectrogram along with the raw LFP and theta signal of single cycle examples. It also shows the prevalence of tSC cycles as defined for the analyses shown in Figures 1 and 2.

Figure S5, related to **Figures 1** and **2.**
Shows that the results displayed in Figures 1 and 2 hold when considering that the tSC strengths of individual theta cycles lie on a continuous, multidimensional spectrum. It also compares nested spectrograms and spike temporal patterns of theta cycles defined by tSC strength to those of theta cycles defined by a standard band-pass filtering approach.

Figure S6, related to **Figure 3.**
Shows speed control analysis for results presented in Figure 3.

Figure S7, related to **Figure 4.**
Shows speed and spatial control analysis for results presented in Figure 4.

Figure S8, related to **Figure 6.**
Shows speed and spatial control analysis, as well as additional spatial and temporal details, for results presented in Figure 6.

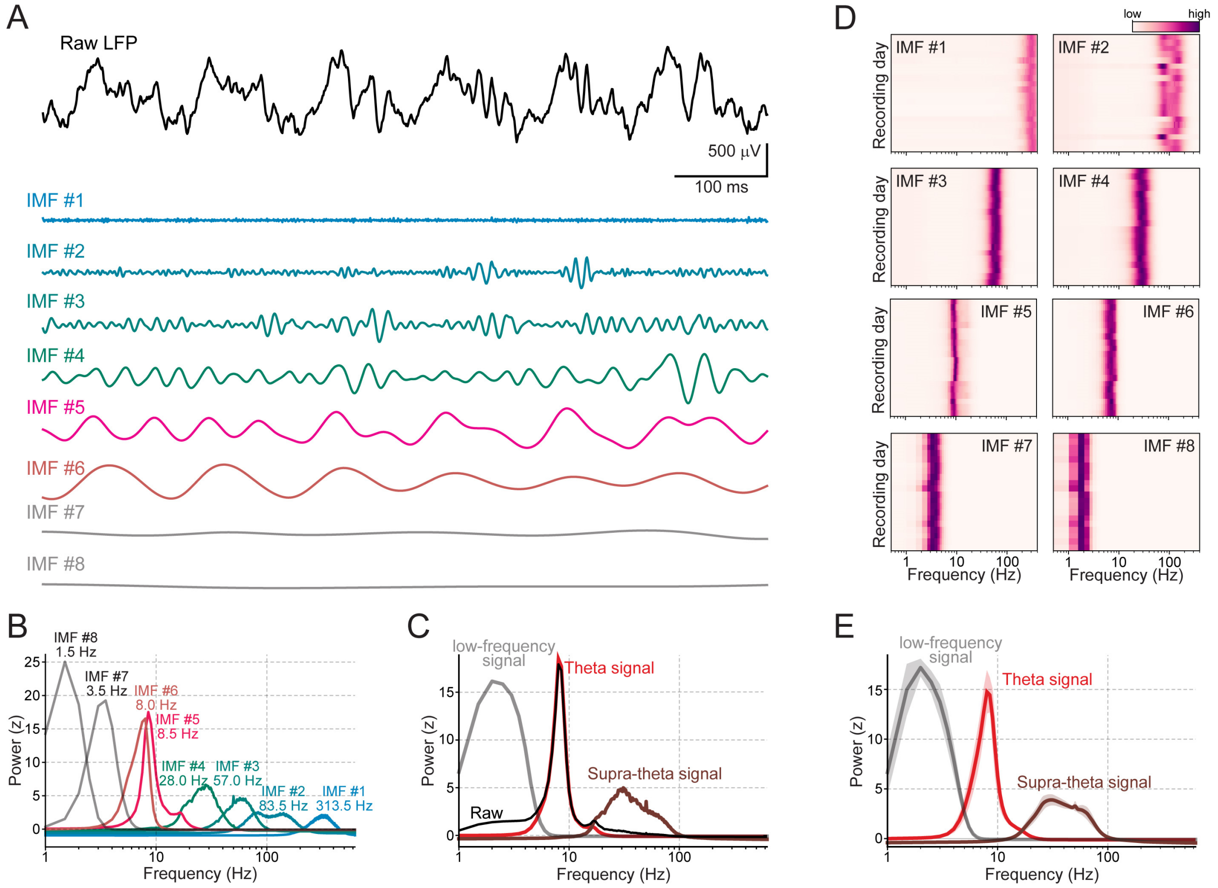

Figure S1. Decomposing LFP signals with EEMD (related to **Figure 1**).
(A) Example of raw CA1 pyramidal layer LFPs (top, black trace) decomposed by EEMD into its Intrinsic Mode Functions (IMFs; color-coded traces).
(B) Power spectrum (z-scored) of each IMF extracted from one recording day (a sample of which is shown in **A**). Numbers indicate the peak frequency of each IMF, as labelled. Note that for visualization purposes only, we normalized each power spectrum individually by setting its mean to zero and standard deviation to one (z-score).
(C) Power spectra (z-scored) of the low-frequency, theta and supra-theta signals shown in **B**. Low-frequency and supra-theta signals were defined as the sum of IMFs with mean frequencies below 5 Hz and above 12 Hz, respectively. The power spectrum obtained for the raw LFP is shown for reference (black line).
(D) Power spectra of the IMFs extracted from CA1 pyramidal layer LFPs in different recording days (n = 20).
(E) Same as **C** but for group data. Shown are mean and one standard deviation across same recording days shown in **D**.

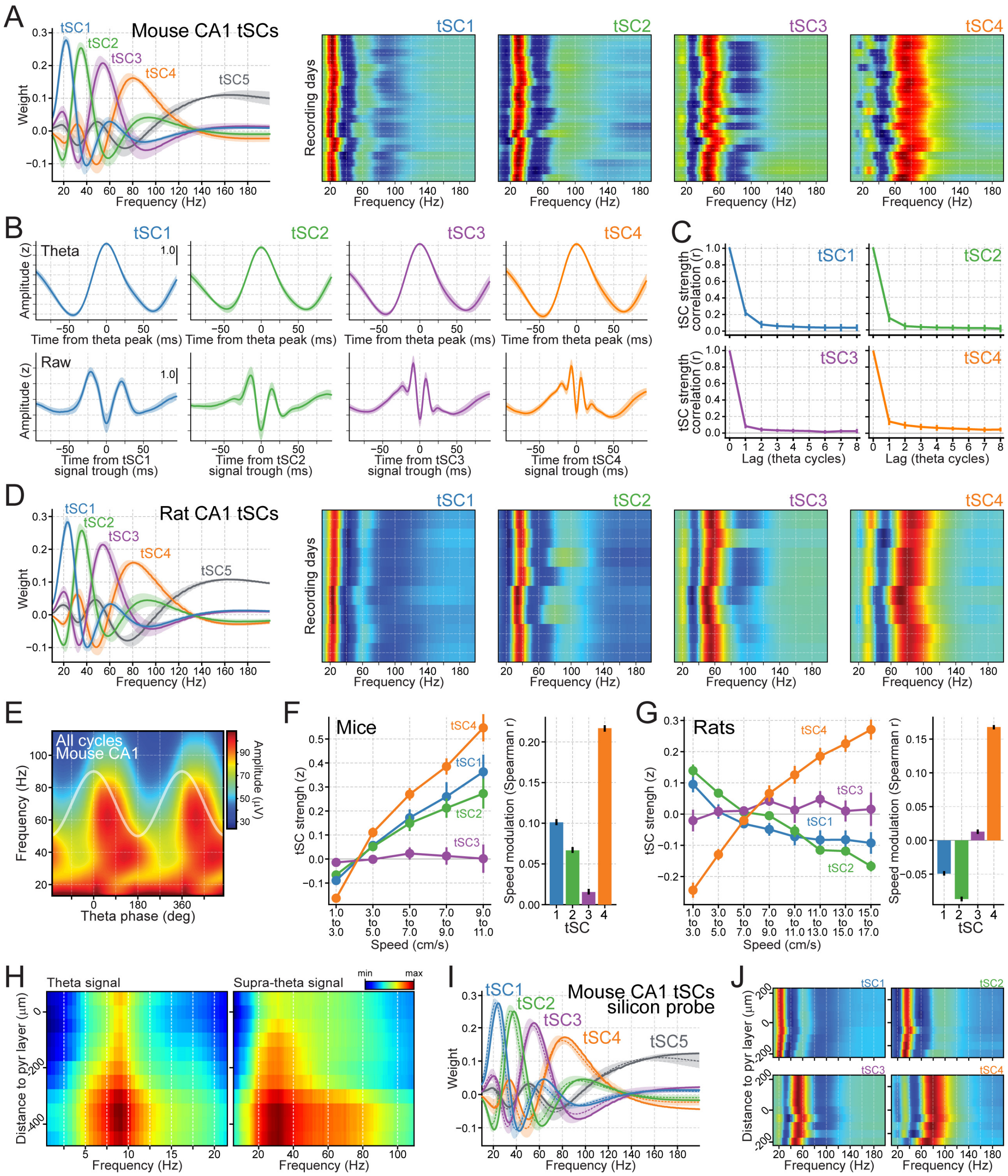

Figure S2. Consistency of theta-nested Spectral Components (tSCs) across recording paradigms (related to **Figure 1** and **Figure 5**).

(A) Left: mean tSCs detected from the mouse dorsal hippocampal CA1 pyramidal layer LFPs recorded using tetrodes (n = 20 recording days of spatial exploration of open-field enclosures; shaded areas correspond to standard deviations). Right: panels show tSC vectors obtained in mouse recording days, as labelled. Peak frequencies across recording days (median and interquartile range) are tSC1: 22Hz, 21–23Hz; tSC2: 35Hz, 34–36Hz; tSC3: 54Hz, 52–55Hz; tSC4: 80Hz, 77–82Hz; tSC5: 169Hz, 153–174Hz.

(B) Top: the mean theta waveform computed for a given tSC. For each recording day, theta signal (z-scored) is averaged around the peak of the cycles assigned to a given tSC. Shown are averages across mouse recording days (with standard deviations shown as shaded areas).

Bottom: same analysis for raw LFPs averaged around tSC signal troughs.
(C) Autocorrelation of tSCs across consecutive theta cycles (mean and standard deviation, n = 20 mouse recording days). Shown are the linear correlations obtained for the strength of a given tSC to itself in *n* subsequent theta cycles (lag in horizontal axis).
(D) Same as in **A**, but for the tSCs detected from the rat dorsal hippocampal CA1 pyramidal layer LFPs recorded using tetrodes (n = 8 recording days from 4 rats during spatial exploration; shaded areas correspond to standard deviations). Peak frequencies across recording days (median and interquartile range) are tSC1: 23Hz, 23–25Hz; tSC2: 36Hz, 34–37Hz; tSC3: 55Hz, 53–55Hz; tSC4: 81Hz, 79–83Hz; tSC5: 162Hz, 160–167Hz.
(E) Same as panel “All cycles” of **Figure 1D**, but with a different amplitude scale for visualization purposes.
(F) Left: z-scored strength of a given tSC (vertical axis) in a given speed bin (horizontal axis) for mouse recordings. In order to control for the unbalance of number of cycles in different speed bins, we used a bootstrap method used by Kemere et al (2013) to compute speed modulation of each tSC. More specifically, (1) for each recording day we randomly sampled a number of theta cycles in each speed range (defined by the less populated bin, typically the one with highest speed); (2) computed the Spearman correlation between speed and tSC across the bootstrapped sample; (3) repeated the procedure 10,000 times to estimate mean and 95% confidence interval. Circles and bars show mean and standard error of the mean (SEM) across the 20 recording days, respectively. Right: corresponding Spearman correlation between speed and tSC strength (mean and bootstrap 95% confidence interval).
(G) Same as in **F**, but for rat recordings (n = 8 recording days).
(H) Power spectra of theta and supra-theta signals extracted from mouse hippocampal CA1 LFPs using different silicon probes contacts and shown as function of depth (with values decreasing from pyramidal to lacunosum moleculare layers).
(I) Same as in **A** but for the silicon probe recordings of the mouse dorsal hippocampal CA1 pyramidal layer LFPs. Pyramidal layer channel defined as the channel with the strongest ripple oscillations. Mean tSCs shown in **A** are displayed for reference (color-coded dotted lines, with associated standard deviations shown as shaded areas).
(J) Theta SCs extracted from mouse hippocampal CA1 LFPs recorded using different silicon probes contacts with depth relative to pyramidal cell layer (defined as the recording site with highest ripple power).

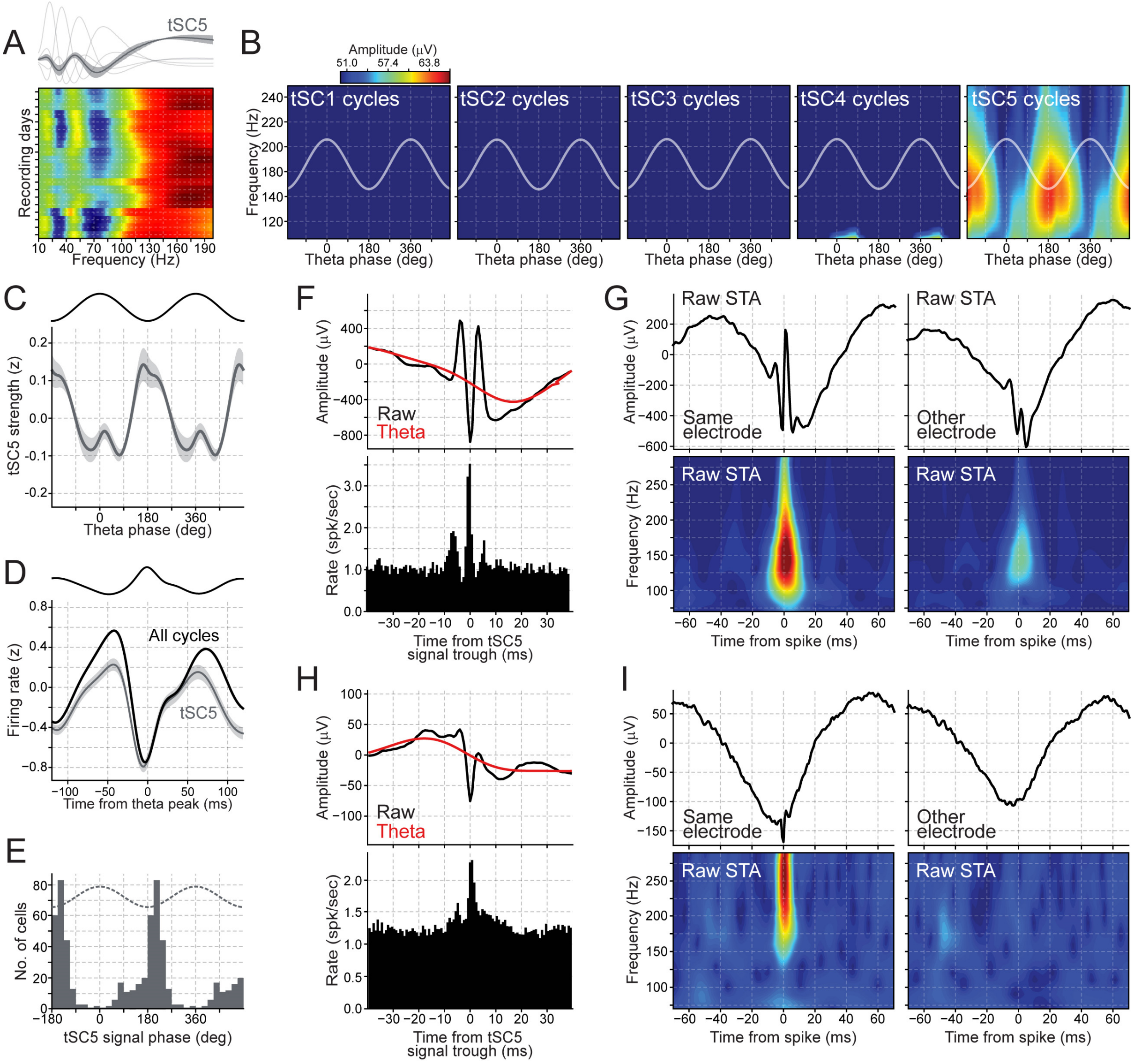

Figure S3. Characteristics of tSC5 (related to **Figure 1** and **Figure 2**).
(A) Top: the mean of tSC5 (n = 20 recording days; with standard deviation as a shaded area). Bottom: corresponding frequency content across all recording days.
(B) Mean amplitude of supra-theta frequencies above 115 Hz computed from the raw LFPs as a function of theta phase. Each panel displays this analysis computed for theta cycles strongly expressing a particular tSC. Cosine indicates theta phase reference with two cycles for clarity.
(C) Strength of tSC5 as a function of theta phase. The strength is computed at each time point as the inner product between tSC5 and the supra-theta signal spectrogram. Theta SC5 strength peak theta phase: median 170º, interquartile range = 158–210º; n = 20 recording days from 10 mice. Shaded areas indicate SEM. Cosine indicates theta phase reference with two cycles for clarity.
(D) Average instantaneous firing rate (z-scored) of principal cells in theta cycles. Results are computed either using all theta cycles or only those strongly expressing tSC5. Spike counts of each neuron are binned with 0.8 ms time windows and smoothed with a 10 ms standard deviation Gaussian kernel. Results shown as means over all principal cells with shaded areas indicating 95% confidence interval. The averaged raw LFP waveform across recording days is displayed on top as a reference.
(E) Distribution of the preferred firing phase of principal cell spikes to tSC5 signal. Dashed cosine indicates tSC5-signal phase reference using two cycles for clarity. Only cells with

significant coupling are included (see Methods).
(F) Top: Average raw LFPs and tSC5 signal triggered by the troughs of the latter. The troughs of the tSC5 signal are detected within its corresponding theta cycles of the corresponding recording day. A single tSC5 signal trough (with the most negative value) per theta cycle was used to avoid auto-correlation distortions. Bottom: mean instantaneous firing rate triggered by the same tSC troughs as in the panel above. Note that the population spiking oscillates around tSC5 signal troughs. This cannot be explained by the autocorrelation of the tSC5 signal since only one tSC5 trough per theta cycle is taken.
(G) Top left: a raw LFP spike-triggered average (STA) from the same recording day shown in **F**. Spikes were taken from theta cycles assigned to tSC5. Spikes and LFP are recorded from the same tetrode. Bottom: the wavelet spectrogram of the STA shown above. Note the theta component as well as a higher frequency component (~100 to 225 Hz) showing that the spikes are coupled to both theta and fast-gamma oscillations. Right panels: the same analysis using the same spikes but for the LFP recorded from a different tetrode. Note that the spike phase coupling to fast oscillations (>100 Hz) is still observed. This strongly suggests that the coupling observed is not an artefact due to spike leakage contamination since the spikes being used were not detected in this LFP channel (Lasztóczi and Klausberger, 2014).
(H) Same as in **F**, but for a different recording day. Note the absence of clear oscillations around tSC5 signal troughs.
(I) Same as in **G**, but for the recording day shown in **H**. Note that when spikes and LFP recorded from same tetrode are used, a waveform resembling a spike is seen in the STA (top left panel). Furthermore, the spectrogram of the corresponding STA (bottom left) shows a non-bounded frequency component for frequencies >~175 Hz. When using an LFP from a different channel (left panels) coupling to fast frequency components are not observed. Taken together, tSC5 signals for this particular recording day seem to be dominated by spike leakage artefacts (Scheffer-Teixeira et al., 2013).

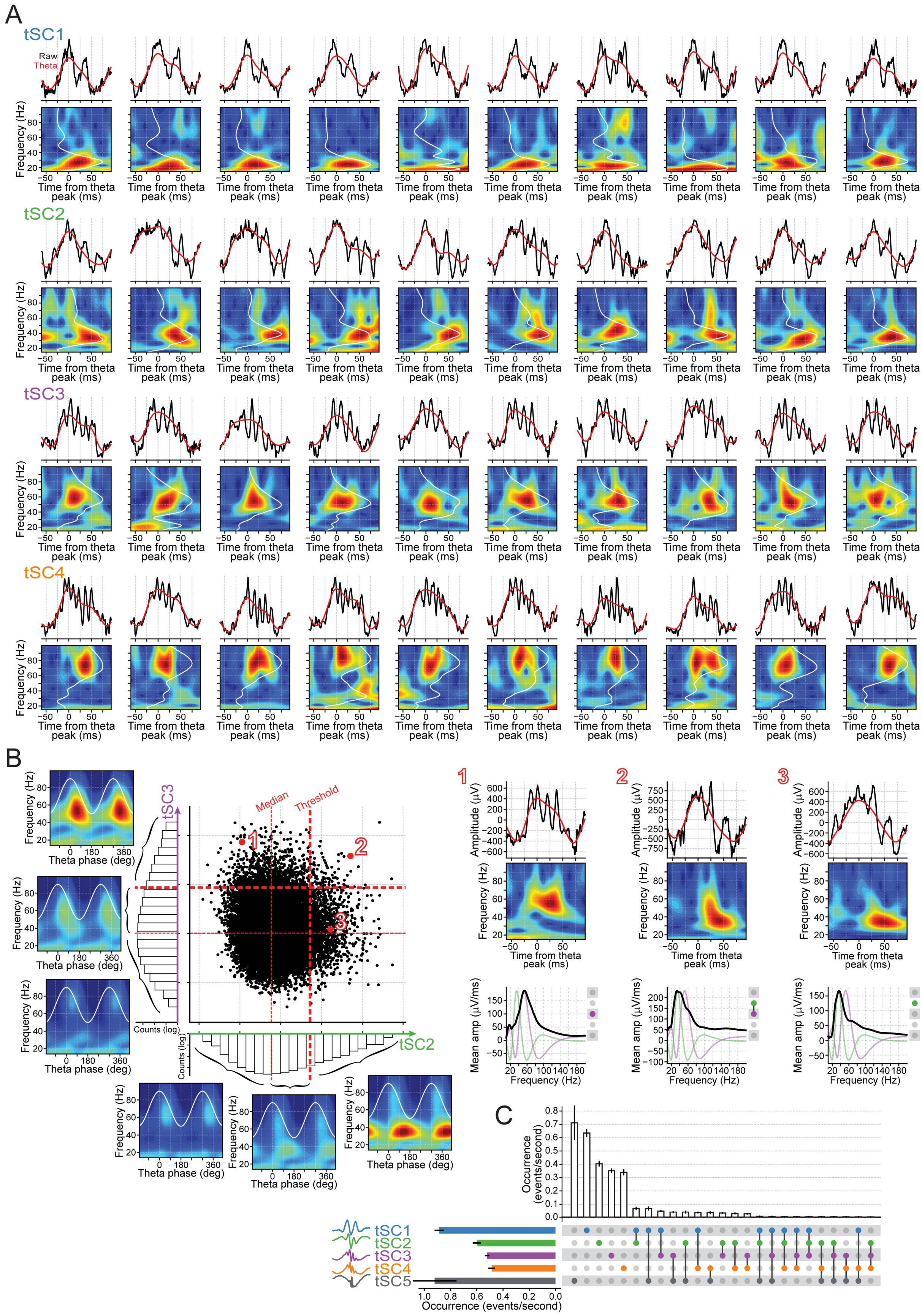

Figure S4. Prevalence of theta-nested spectral profiles (related to **Figure 1** and **Figure 2**). (A) Examples of individual theta cycles strongly expressing a given tSC (i.e., strength above

threshold; see STAR methods). All examples are from the same mouse as in **Figure 1E**.
(B) Left: Scatter plot shows the theta cycles of one recording day projected in the subspace spanned by tSC2 and tSC3. Each individual theta cycle is represented as a data point defined by its tSC2 (horizontal axis) and tSC3 (vertical axis) strength. Marginal distributions are shown along each axis. Dashed lines indicate median value of marginal distributions (thinner) and thresholds for assigning cycles to a tSC (thicker). Panels along each marginal distribution show the same analysis as **Figure 1D**, but for different ranges of tSC strength (below median, between median and threshold, and above threshold), as indicated. Note that the strength of the tSCs does not reveal non-overlapping clusters of theta cycles but instead lies on a multidimensional continuum. Right: Examples of three single cycles (indicated by numbers 1, 2, 3) from the scatter plot shown on the left. Bottom panels show the corresponding spectral signatures (black) along with the tSC2 (green) and tSC3 (purple) signals.
(C) UpSet plot (Lex et al., 2014) showing the occurrence (events per second) of theta cycles strongly expressing a tSC (as defined by the threshold shown in **B**) or a combination of tSCs (mean and 95% confidence interval, n = 20 recording days).

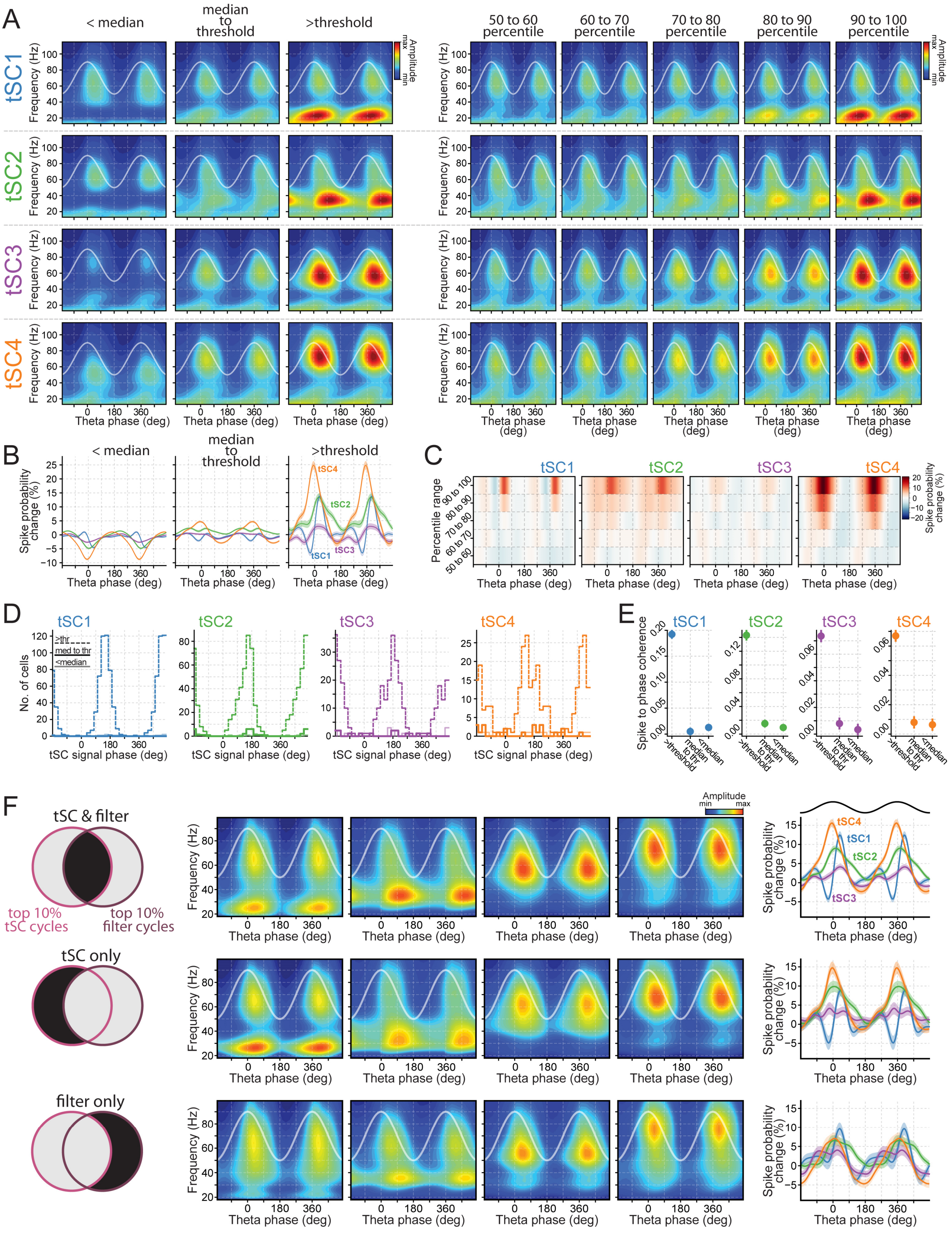

Figure S5. Relation between firing modulation of principal cells and tSC strength (related to **Figure 1** and **Figure 2**).

(A) Mean amplitude of supra-theta frequencies computed from the raw LFPs as a function of theta phase (same analysis as **Figure 1D)** and for different subsets of theta cycles sliced on the basis of tSC strengths. Left: data slices corresponding to theta cycles with tSC strength below median, between median and threshold, and above threshold (see **Figure S4B**); Right: data slices corresponding to 10-percentile data slices of tSC strength, as indicated. All

spectrograms were computed from z-scored LFP traces before being averaged for different theta phases in order to combine data from different mouse recording days (n = 20).
(B) Change in spike probability of principal cells as a function of ongoing theta phase (mean ± SEM). Changes were computed as in **Figure 2C** (relative to the grand-average computed from all theta cycles) for theta cycles with tSC strength below median, between median and threshold, and above threshold. Rightmost panel shows same results as **Figure 2C**, for reference. Note that the firing modulation seen in theta cycles nesting a tSC signal above threshold (Rightmost panel and **Figure 2C**) is greatly diminished in the other slices.
(C) Same analysis as in **B** but for the 10-percentile data slices of tSC strength. Note that the strong spike modulation is only seen in the top slices.
(D) Distribution of the preferred firing phase of principal cell spikes to tSC signals (same analysis as in **Figure 2E**) for theta cycles with tSC strength below median, between median and threshold, and above threshold. Dashed lines represent results shown in **Figure 2E**, for reference. As before, 0 and 180 phases correspond to peak and trough, respectively. Note that the significant proportion of neurons coupled to the phase of strong tSC signals (tSC strength above threshold; dashed lines and **Figure 2E**) is drastically diminished in the other slices. Of note, we also found that a significant proportion of neurons coupled to the phase of multiple tSC signals in theta cycles nesting more than one strong tSC. Namely, more than 60% of neurons significantly coupled to tSC1 or to tSC2 also coupled to another given tSC in cycles expressing both tSCs (62, 64 and 66% of tSC1-coupled neurons also coupled to tSC2, tSC3 and tSC4, respectively; and 81, 64, 60% of tSC2-coupled neurons also coupled to tSC1, tSC3 and tSC4). This was less pronounced for tSC3- and tSC4-coupled neurons (19, 53 and 58% of tSC3-coupled neurons also coupled to tSC1, tSC2 and tSC4; and 15, 36, 67% tSC4-coupled neurons also coupled to tSC1, tSC2 and tSC3).
(E) Spike-to-tSC-signal phase coherence (mean ± 95% confidence interval) obtained for theta cycles with tSC strength below median, between median and threshold, and above threshold. See STAR methods for details.
(F) Spectrograms and spike temporal patterns nested in theta cycles defined by tSCs or band-pass filters. We evaluated whether the tSC strength (quantified by the projection of single theta cycle spectral signatures onto a tSC axis) could be replaced by the envelope of LFP signals band-pass filtered to the corresponding tSC frequency range. For that, we designed 2nd order Butterworth band-pass filters matching the frequency band of each tSC (tSC1: 18-25Hz; tSC2: 31-40Hz; tSC3: 48-61Hz; tSC4: 71-93Hz). These bands were defined from tSC vectors: starting from the peak frequency, we iteratively increased the range until they included at least 75% of the total norm of the tSC vector. For each recording day, we used the top 10% theta cycles defined in terms of tSC strength or in terms of filtered energy (more specifically, computing the mean Hilbert envelope of the supra-theta signal filtered by the tSC corresponding band-pass filter). Therefore, apart from the tSC strength (replaced by the filtered energy for the purpose of this comparison), all remaining analysis steps were exactly the same (and as before). Top panels show mean spectrograms (as in **A**) of cycles found both by the tSC strength and band-pass filter approaches, along with the same principal cell rate analysis as in **B** and **C** but for these cycles (only cells with at least 250 total spikes in these cycles were included). Note that these cycles show similar spike temporal patterns as those shown in **B**, **C** and **Figure 2C**. Mid panels display the results obtained for the theta cycles found by the tSC method but not by the filter-based approach. Bottom panels show results obtained for cycles found by the filter approach but not by the tSC approach. Note that the tSC strength measure is not exactly equivalent to the power measured using simple filters. This is because each tSC weight vector represents a specific pattern of spectral content, as opposed to merely the amount of energy within a defined frequency range. Such a tSC spectral pattern notably allows capturing the contrast between a frequency band and its

neighbouring frequencies. This could in principle prevent, for example, broad frequency increases (such as elevated 1/f events) to influence tSC strength. We found that theta cycles assigned to both a given tSC (by the tSC approach) and its corresponding band (by the filter approach) successfully retrieved the expected spectrograms and spike temporal patterns (as shown in panel **A**). We observed similar results for the theta cycles found by the tSC projection approach only (i.e., not by the filter approach). This indicated that some cycles (with similar activity patterns) were missed by the band-pass detection. Finally, the cycles detected exclusively by the filter-based method showed more smeared frequency components and weaker spike correlates. These results suggest that band-pass filtered energies could be more vulnerable to interferences of broad-frequency events or by components partially overlapping in frequency with the band of interest. We noted, however, that the intersection between the two groups of theta cycles was typically larger than each “exclusive” set of theta cycles. It is therefore possible that the correlates of tSCs could be replicated by further refinements in the use of filters, including the design of more complex transfer functions.

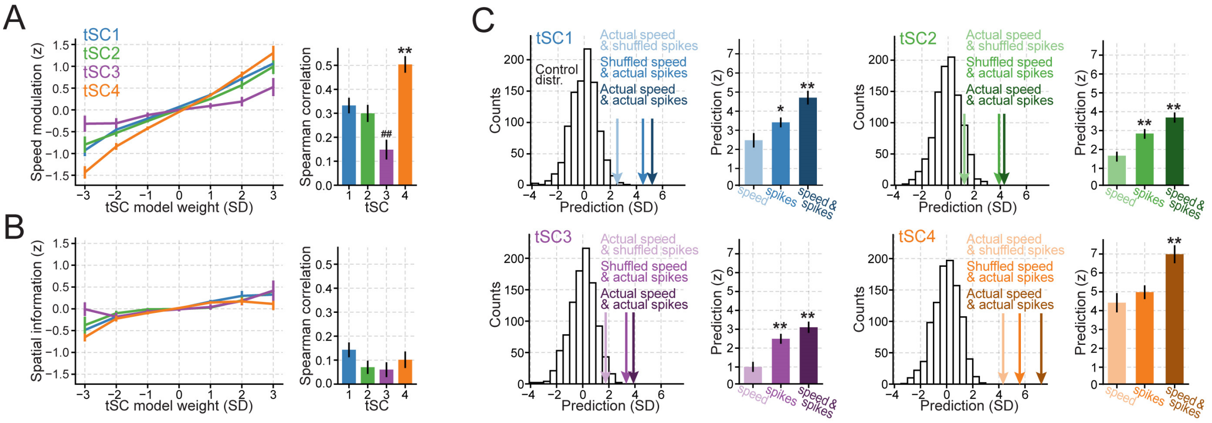

Figure S6. Theta Spectral Components GLM supplementary information (related to **Figure 3**).

(A) Left: relationship between tSC GLM weights attributed to individual principal cells and their speed modulation. To allow for a comparison of the tSC GLMs obtained across recording sessions, the weights obtained in each tSC model were divided by the standard deviation calculated for weights of that model. This normalization was done to combine data from recording days with different number of recorded neurons. Speed modulation of a neuron was quantified as the Spearman correlation between its spike counts and speed values measured across theta cycles. Shown are averages and SEM across 20 recording days. Right: Spearman correlation (mean ± SEM) between tSC model weights and speed modulation for different tSCs. Note that the tSC4 correlation is significantly higher than that of tSC1 ($p = 0.0018$), tSC2 (0.00012) and tSC3 ($p < 10^{-7}$); further, tSC3 correlation is significantly lower than tSC1 ($p = 0.0007$) and tSC2 ($p = 0.008$). Two-way ANOVA with Tukey multiple comparisons of means.

(B) Same as in **A**, but for spatial information instead of speed modulation (all p values > 0.15; two-way ANOVA with Tukey multiple comparisons of means).

(C) Histograms display examples of control distributions obtained for GLM predictions by circularly shuffling across theta cycles both speed and spike content together in relation to tSC strength. Prediction values are expressed as distances from the mean of such control distributions in terms of control distribution standard deviations (as in **Figure 3C**, left). Arrows show predictions obtained by re-computing the GLMs and their prediction performance shown in **Figure 3** in three conditions where the data from each theta cycles corresponded to either: (1) the original speed with shuffled spikes (i.e., spikes coming from another cycle using a random circular shift of spike counts across theta cycles), (2) the original spikes with shuffled speed (i.e., same as before but with circular shift of speed values across theta cycles), or (3) the original speed with original spikes, as color coded. Bar plots display group results for each tSC, as labelled. See STAR methods for more details. Note that the third condition, which keeps the original relationship between spikes and theta cycles, remarkably increased the prediction power above that of the first condition where the models are based on actual speed with spike content of other theta cycles (tSC1: $p < 6.6 \times 10^{-10}$; tSC2: $p < 1.2 \times 10^{-9}$; tSC3: $p < 8.3 \times 10^{-10}$; tSC4: $p < 5.5 \times 10^{-10}$; Wilcoxon test). Remarkably, for predicting tSC1, tSC2 and tSC3 strength, we found that spike content of individual theta cycles conveyed more information to GLMs than speed, as indicated by the higher performance of models trained with actual spike counts compared to the ones trained with actual speed ($p = 0.003$, $p = 0.0002$, $p < 3.9 \times 10^{-8}$, respectively. Wilcoxon test). A similar trend was observed for tSC4, although non-significant ($p = 0.12$), likely due a ceiling effect as tSC4 was more strongly modulated by speed than the other tSCs (**Figure S2F**).

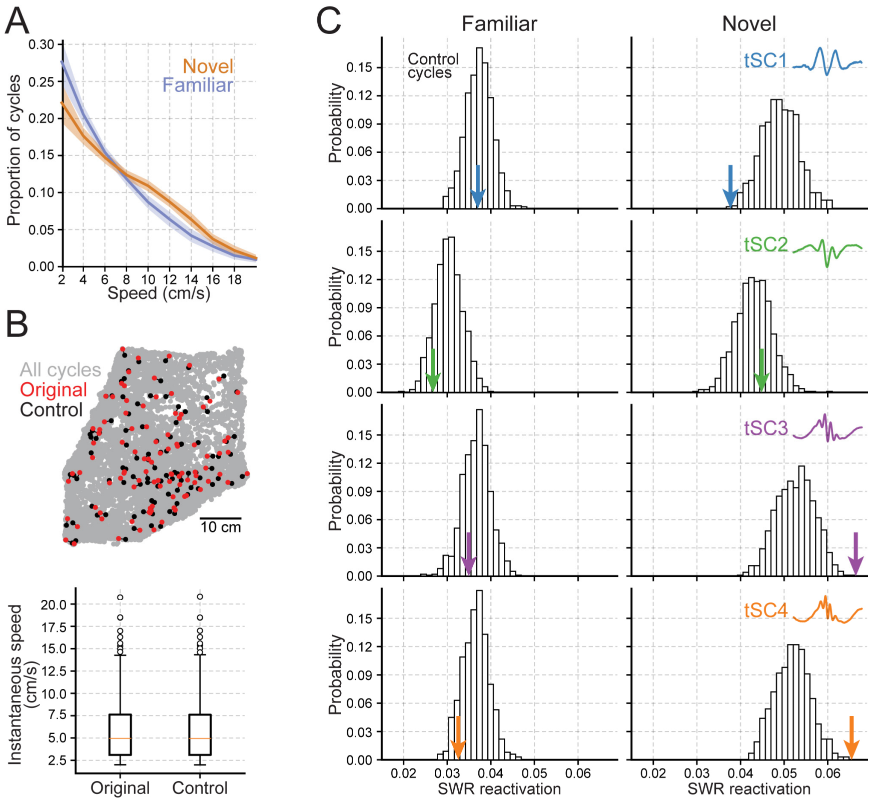

Figure S7. Speed and location distributions do not account for the enhanced offline SWR reactivation observed for theta cycles with strong tSC3 or tSC4 (related to **Figure 4**).

(A) Proportion of instantaneous speed for theta cycles detected in the mouse CA1 pyramidal layer LFPs during exploration of familiar and novel enclosures (mean ± 95% confidence interval).

(B) Example of speed- and location-matching control for theta cycles. Top: location of theta cycles assigned to tSC4 (red dots) and their matched controls (black dots) randomly selected from a 5-cm radius from the originals and similar instantaneous speeds (< 0.5 cm/s difference from originals). Location of all cycles in the same recording session represented by gray dots. Bottom: corresponding distributions of instantaneous speed for both original and control cycles. See STAR methods for more details.

(C) Offline SWR reactivation following exploration of familiar and novel environments using the same analysis as in **Figure 4B** but calculated from control distributions (n = 1000) with randomly selected theta cycles to match speed and location of theta cycles originally assigned to each tSC (as shown in **B**). For this analysis, each control was computed by replacing each original theta cycle by another one with similar speed (no more than 0.5 cm/s difference) and occurring at a nearby location (no more than 5 cm away). Thus, each control was composed by a set of cycles with virtually the same speed and spatial distributions. Note that this analysis replicates enhanced SWR reactivation of tSC3 and tSC4 (both tSC1 and tSC2: $p > 0.3$; both tSC3 and tSC4: $p < 0.001$) following exploration of novel enclosures. Thus, even when compared to theta cycles matched by animal speed and location, the firing correlation structure in cycles nesting tSC3 and tSC4 predicts reactivation in post-exploration SWR co-firing more accurately.

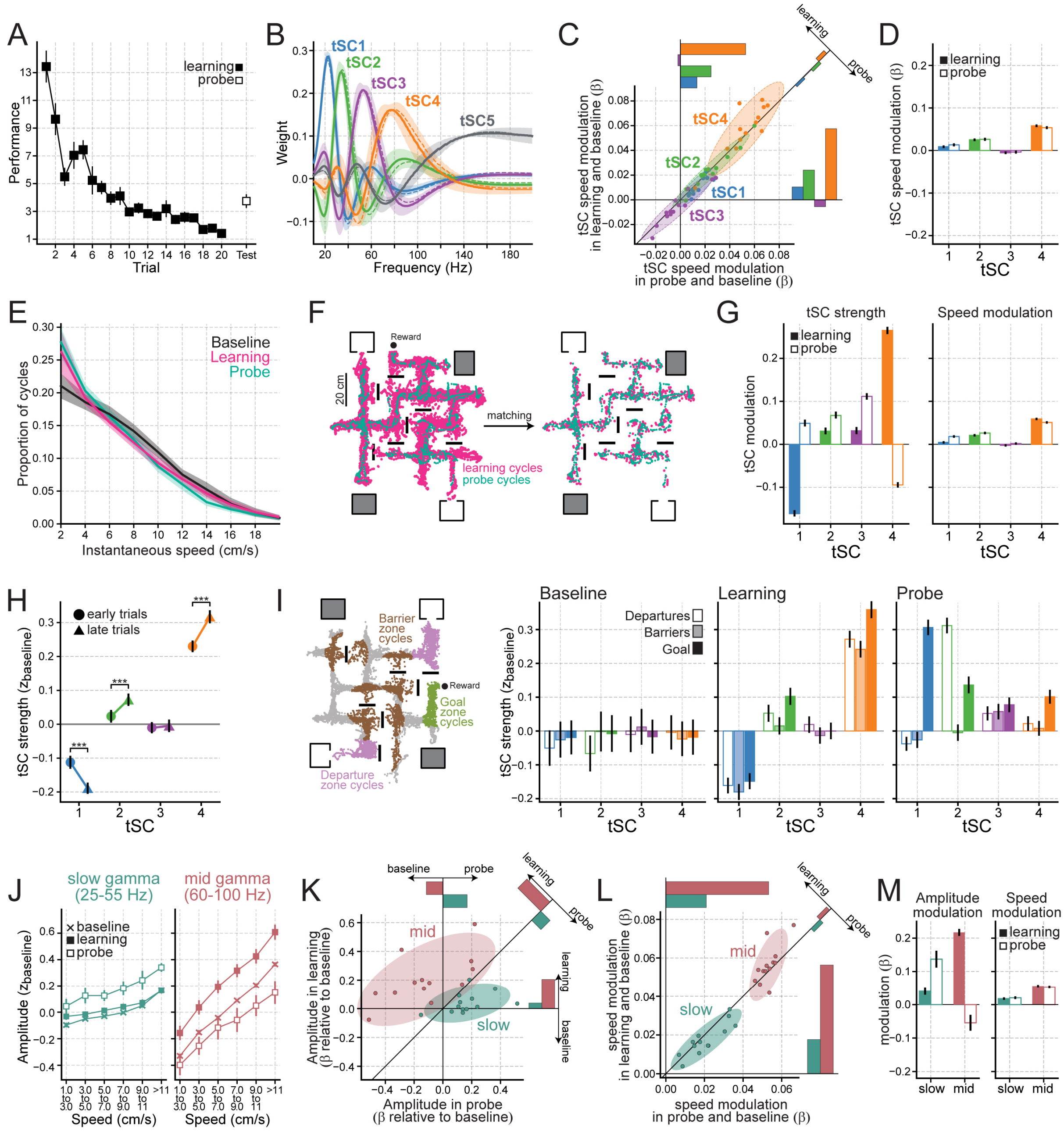

Figure S8. Crossword maze supplementary information (related to **Figure 6**).
(A) Behavioral performance during learning trials and memory probe test of the crossword maze task (mean ± SEM; n = 13 recording days from 6 mice). Performance was measured as the distance travelled in each learning trial or the probe test, divided by the length of the optimal (shortest) path from the departure box to the reward location in use.
(B) Theta SCs identified in the crossword maze tetrode dataset (solid lines and shaded areas represent mean and standard deviation across recording days). For comparison, dashed lines show mean tSCs extracted in the open-field tetrode dataset (see **Figures 1C** and **S2**).
(C) Theta SC speed modulation during learning and probe stages. Each dot represents the tSC speed modulation during learning trials (vertical axis) and memory probe test (horizontal axis) for a given recording day, and relative to the baseline. Diagonal solid black trace shows y=x line along which the speed modulation of a tSC strength in learning and probe test is equal. Each bar plot represents the average projection of a (color-coded) tSC in that axis.

Shown are speed modulations for the same ANOVA model regression used to compute results in **Figure 6C** (see STAR methods for details).
(D) Theta SC speed modulation by task stages. Shown are speed modulations for the same ANOVA model regression used to compute results in **Figure 6D** (see STAR methods for details). Error bars represent 95% confidence interval.
(E) Proportion of instantaneous speed for theta cycles detected during the baseline, learning and probe stages (mean ± 95% confidence interval).
(F) Example of matching control between theta cycles of probe and learning stage. For this analysis, cycles from the learning and baseline stages were randomly selected to match the speed and location of theta cycles from the probe, as the latter stage always contained fewer theta cycles (i.e., shorter recording session). More specifically, for each theta cycle in a probe session, we randomly selected one from learning (or baseline) with similar speed and location (no more than 0.5 cm/s speed difference and 5 cm away).
(G) Theta SC modulation by task stages using the same analysis as in **Figures 6D** and **S6D** but for speed- and location-matching controls. Bars display mean ANOVA coefficients obtained from 20000 bootstrap controls (bars show bootstrap 95% confidence interval).
(H) Mean strength of different tSCs for the first and last learning trials. For this analysis, the spatial and speed distribution of the first three trials was matched to the last three trials, as the latter trials were of shorter duration. More specifically, for each theta cycle detected in the last trials group, we randomly selected one from the first trials with similar speed and location (no more than 5 cm away and 0.5 cm/s speed difference). Shown are the means across 20,000 of such controls (bars show bootstrap 95% confidence interval).
(I) Left panel shows theta cycles (color-coded dots) occurring in the departure, barrier and goal zones, as labelled; remaining theta cycles (outside of these zones of interest) are shown in gray. Right panels show z-scored tSC strengths for theta cycles observed in each zone and for different task stages. Note that tSC strength values were z-scored with respect to baseline (as in **Figure 6**). Theta cycles in each zone were speed-matched to the distribution observed in the goal zone. See STAR methods for more details.
(J) Amplitude of predefined frequency bands for (25–55Hz) slow-gamma and (60–100Hz) mid-gamma oscillations as a function of task stages and locomotion speed (mean ± SEM across recording days). See results in **Figure 6B** for comparison with unsupervised detection of LFP frequency components.
(K) Amplitude modulation of 25–55Hz slow-gamma and 60–100Hz mid-gamma bands filtered from LFPs (as in **J**) during learning and probe stages relative to baseline, following same procedure as in **Figure 6C**. Each dot represents the amplitude modulation during learning (vertical axis) and memory probe test (horizontal axis) for a given recording day, and relative to the baseline. Diagonal solid black trace shows y=x line (i.e., along which the strength of a predefined frequency band in learning and probe test would be equal). Each bar plot represents the average projection of the data in a given axis. See results in **Figure 6C** for comparison with unsupervised detection of LFP frequency components.
(L) Speed modulation of 25–55Hz slow-gamma and 60–100Hz mid-gamma bands during baseline and learning/probe stages. Each dot represents the speed modulation during learning (vertical axis) and memory probe test (horizontal axis) for a given recording day, and relative to baseline. Diagonal solid black trace shows y=x line along which the speed modulation in learning and probe test is equal. Compare with **Figure S8C**.
(M) Amplitude and speed modulation of 25–55Hz slow-gamma and 60–100Hz mid-gamma by task stages. Shown are mean amplitude and speed modulations quantified by an ANOVA model regression as in **Figures 6D** and **S8D**, but for corresponding predefined bands. Error bars represent 95% confidence interval.
